# The γ-Core Motif Peptides of AMPs from Grasses Display Inhibitory Activity against Human and Plant Pathogens

**DOI:** 10.3390/ijms23158383

**Published:** 2022-07-29

**Authors:** Marina P. Slezina, Ekaterina A. Istomina, Ekaterina V. Kulakovskaya, Tatyana V. Korostyleva, Tatyana I. Odintsova

**Affiliations:** 1 Vavilov Institute of General Genetics RAS, 119333 Moscow, Russia; omey@list.ru (M.P.S.); mer06@yandex.ru (E.A.I.); tatkor@vigg.com (T.V.K.); 2 Federal Research Center “Pushchino Scientific Center for Biological Research of the Russian Academy of Sciences”, G.K. Skryabin Institute of Biochemistry and Physiology of Microorganisms RAS, 142290 Pushchino, Russia; ekaterina.kulakovskaya@gmail.com

**Keywords:** plant antimicrobial peptides, γ-core, antimicrobial activity, novel antimicrobials, *Fusarium* species, plant pathogenic bacteria, human pathogenic yeasts

## Abstract

Antimicrobial peptides (AMPs) constitute an essential part of the plant immune system. They are regarded as alternatives to conventional antibiotics and pesticides. In this study, we have identified the γ-core motifs, which are associated with antimicrobial activity, in 18 AMPs from grasses and assayed their antimicrobial properties against nine pathogens, including yeasts affecting humans, as well as plant pathogenic bacteria and fungi. All the tested peptides displayed antimicrobial properties. We discovered a number of short AMP-derived peptides with high antimicrobial activity both against human and plant pathogens. For the first time, antimicrobial activity was revealed in the peptides designed from the 4-Cys-containing defensin-like peptides, whose role in plant immunity has remained unknown, as well as the knottin-like peptide and the C-terminal prodomain of the thionin, which points to the direct involvement of these peptides in defense mechanisms. Studies of the mode of action of the eight most active γ-core motif peptides on yeast cells using staining with propidium iodide showed that all of them induced membrane permeabilization leading to cell lysis. In addition to identification of the antimicrobial determinants in plant AMPs, this work provides short candidate peptide molecules for the development of novel drugs effective against opportunistic fungal infections and biopesticides to control plant pathogens.

## 1. Introduction

Pathogenic microorganisms cause diseases in plants and animals. About 80,000 plant diseases have been reported in the world [1]. The overwhelming number of plant pathogens (over 80%) belong to phytopathogenic fungi [2]. Virtually all plants are hosts to particular fungal pathogens. The disease symptoms caused by fungi vary and include spots, wilts, rots, blight, and cankers. The ubiquitous *Fusarium* genus comprises approximately 300 species/species complexes that cause diseases of almost all economically important plants [3]. The most common diseases induced by these pathogens are vascular wilts and root rots. In addition to reducing crop yields, the *Fusarium* fungi produce mycotoxins, toxic secondary metabolites, which contaminate food and thus are harmful to human health. Some of the *Fusarium* species belong to opportunistic human pathogens affecting individuals with impaired immunity [4].

Pathogenic bacteria pose another threat to plants’ health. Of the more than 7000 bacterial species, about 150 species cause diseases in plants [5]. The disease symptoms are diverse: galls, leaf spots, wilts, blights, soft rots, scabs, and cankers. The bacterial species that are pathogenic to plants belong to a number of genera, including *Pectobacterium*, *Pseudomonas*, and *Clavibacter* [6].

The economic consequences of plant diseases for agriculture are yield losses amounting to 50% in epiphytotic years and a reduction in the quality of agricultural production due to its contamination with pathogen-derived toxic secondary metabolites [7]. The use of fungicides reduces yield failure; however, this has a negative impact on the environment and stimulates the emergence of resistant strains of pathogens. With the world’s constantly growing population, the need for new alternative crop protection products is steadily increasing.

About 200 fungal species cause diseases (mycoses) in humans. The genus *Candida* consists of approximately 200 yeast species. Of them, about a dozen are associated with the vast majority of human infections caused by fungal pathogens [8]. In the last two decades, the incidence of human diseases caused by *Candida* species has been steadily increasing [9]. *Cryptococcus neoformans* is another important opportunistic human pathogen [10]. In severe cases, it causes cryptococcal meningitis, an extremely serious infection with high mortality rates. With considerable progress in cancer treatment and organ transplantation, the number of patients with profound immunosuppression, which are at risk of acquiring life-threatening fungal infections, is constantly increasing. The therapeutic efficacy and clinical applicability of the available antifungal agents is limited by their toxicity, narrow activity spectrum, inability to fully eliminate infection, and resistance development. The search for novel alternative antifungals with enhanced potency, minimal toxicity, broad-spectrum activity and effective tissue penetration has become a priority.

Antimicrobial peptides (AMPs) are ubiquitous proteinaceous defense compounds, which comprise an essential part of the immune system of plants, animals, and microorganisms [11,12,13]. The array of AMPs that are present permanently in each plant species and those synthesized in response to pathogen invasion form the basis for the so-called innate (or non-specific) immunity. Plant AMPs are mainly cysteine-rich molecules, whose structure is stabilized by three to five disulfide bonds [14,15,16]. They differ significantly in their amino acid sequences and 3D structure resulting in a multifaceted mode of action and diverse host range. Disturbance of the membranes of pathogenic microorganisms is believed to be the predominant mechanism of microbe killing. As mentioned above, the development of resistance to conventional antibiotics in pathogens and the negative environmental impact of chemicals used for plant protection require the search for new alternative means of fighting human, animal, and plant diseases. Plant AMPs, which are natural antibiotics acting rapidly on a wide range of pathogens and showing a low incidence of resistance development, seem excellent candidates for creating new drugs and plant disease control agents. However, the size of some plant AMPs and their disulfide-linked structures, resulting in high cost of production, are a barrier to their widespread use. To overcome these limitations, short peptide fragments corresponding to the sequences in the intact AMP molecules that are responsible for the antimicrobial activity can be regarded as cost-effective templates for creating novel antimicrobial agents with a view to their use in agriculture and medicine.

Studies on the structure–function relationship in plant AMPs have shown that antimicrobial activity is associated with a specific part of the cysteine-rich peptide (CRP) molecule, the so-called γ-core. γ-Core was defined as a region with a GXCX_3-9_C signature adopting a β-hairpin conformation in the AMP’s 3D structure [17]. In our previous work, we explored the antimicrobial activity of the γ-core motif peptides of a number of *Solanum lycopersicum* L. CRPs involved in response to infection with the fungus *Fusarium oxysporum* and the biogenic resistance inducers [18]. We showed that the γ-cores of several tomato CRPs possess potent antimicrobial activity against plant and human pathogens. In this work, we continue our research on antimicrobial properties of the γ-core motif peptides from plant AMPs. This time, we focused on the AMPs of the wheat species *Triticum kiharae* Dorof. et Migush., which is highly resistant to plant pathogens and is, therefore, potentially a rich source of novel antimicrobials. In this study, we have identified the γ-core motifs in AMPs predicted in the transcriptomes of *T. kiharae* infected with *F. oxysporum* and treated with *F. sambucinum* elicitors, produced them by solid-phase synthesis and assayed their antimicrobial properties against nine pathogens, including yeasts affecting humans, as well as plant pathogenic bacteria and fungi. As a result, we discovered a number of short AMP-derived peptides with high antimicrobial activity both against human and plant pathogens. For the first time, antimicrobial activity was revealed in the peptides derived from the 4-Cys-containing defensin-like (DEFL) peptides, whose role in plant immunity has remained unknown. In addition to identification of the determinants of the antimicrobial properties of plant AMPs, this work provides short candidate peptide molecules for the development of novel drugs effective against opportunistic fungal infections and biopesticides to be used in control of plant pathogens.

## 2. Results

### 2.1. Design of γ-Core Peptides

**Figure 1:**
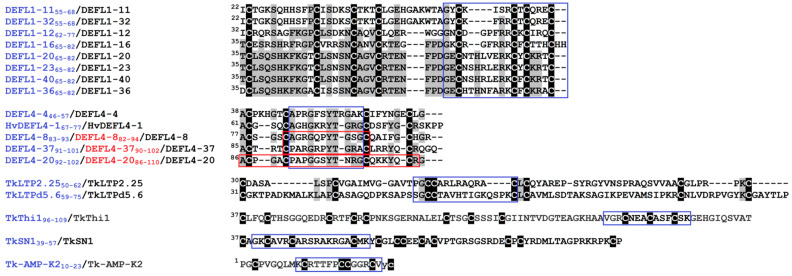
Multiple sequence alignment of the γ-core-containing regions of selected wheat and barley AMPs. Superscript numbers denote their position in the precursor proteins. The names of synthetic peptides and their parent AMPs are shown on the left. The sequences of synthesized peptides are framed (in purple and red). Cysteine residues are shaded black, and identical amino acids are shaded gray.

Seventeen AMPs belonging to the families of DEFLs, snakins, non-specific lipid-transfer proteins (nsLTPs), thionin- and knottin-like peptides, which were identified in wheat by transcriptome or amino acid sequencing [19,20,21,22], and peptide HvDEFL4-1 of barley discovered by BLAST search (GenBank: BAJ88142.1) were selected for the synthesis of the γ-core motif peptides and their truncated variants (Figure 1). The γ-core peptides and their derivatives were produced by chemical synthesis and purified by high-performance liquid chromatography.

#### 2.1.1. Defensin-like Peptides

##### 8-Cys DEFLs

Eight γ-core motif peptides (DEFL1-11_55-68_, DEFL1-12_62-77_, DEFL1-16_65-82_, DEFL1-20_65-82_, DEFL1-23_65-82_, DEFL1-32_55-68_, DEFL1-36_65-82_ and DEFL1-40_65-82_) were designed from the sequences of classical defensins: DEFLs 1-11, 1-12, 1-16, 1-20, 1-23, 1-32, 1-36 and 1-40, which have a characteristic 8-Cys motif C-X{4,25}-C-X{2,12}-C-X{3,4}-C-{3,17}-C-X{4,32}-C-X-C-X{1,6}-C [19]. All selected DEFL genes were up-regulated in the plants displaying induced resistance (IR) [19], which suggested their involvement in the phenomenon. The synthetic γ-core peptides of the classical defensins in addition to the γ-core motif GXCX_n_C contained a C-terminal region including two additional cysteine residues (Figure 1). Several γ-core peptides showed high sequence similarity that allowed us to identify the residues essential for antimicrobial activity against particular pathogens. For example, DEFL1-23_65-82_ and DEFL1-40_65-82_ differed by a single residue (Y13F) between the second and the third cysteines. DEFL1-11_55-68_ differed from DEFL1-32_55-68_ by two amino acid residues: the IS sequence in DEFL1-11_55-68_ was substituted by FR in DEFL1-32_55-68_. The γ-core peptides DEFL1-20_65-82_ and DEFL1-23_65-82_ were also very similar, differing in three amino acid residues in the loop between the first two cysteines, two of which were conserved substitutions. The DEFL1-16 γ-core DEFL1-16_65-82_ possessed a highly basic hexapeptide RGFRRR, which was discovered in a number of defensins found in plants of different families [23].

##### 4-Cys DEFLs

Four synthetic peptides were designed on the basis of the sequences of 4-Cys-containing DEFLs, which have a cysteine signature as follows: C-X{3,5}-C-X{8,17}-C-X{4,6}-C (Figure 1). When compared to the classical defensins, the biological functions of 4-Cys-containing DEFLs in wheat physiology remain obscure [19], therefore, testing the antimicrobial properties of these CRPs seemed a reasonable way to elucidate their role in defense. The selected DEFLs included DEFL4-4, DEFL4-8, DEFL4-20 and DEFL4-37. All but one 4-Cys DEFL showed sequence similarity to uncharacterized proteins of the diploid relatives of hexaploid wheat—*Aegilops tauschii* Coss. and *Triticum urartu* Thumanjan ex Gandilyan [19]. DEFL4-4 displayed no sequence similarity to known proteins. All selected 4-Cys DEFLs except DEFL4-37 possessed a γ-core motif and were predicted to be AMPs. The expression profiling showed that the DEFLs 4-4, 4-8 and 4-20 were up-regulated by the elicitors and/or fungal infection suggesting their involvement in the activation of the immune response by the resistance inducers and/or pathogen [19]. Conversely, DEFL4-37 was down-regulated, pointing to its role as a negative regulator of the immune response in wheat. In addition to the full-length DEFL4 γ-core peptides, their shortened versions were also synthesized (Figure 1). Truncated variants of the γ-core peptides with high antimicrobial activity are preferable for practical applications due to a low cost of production. These truncated versions of DEFL4 γ-core peptides represented the loops between the second and the third cysteine residues. The avoidance of highly reactive cysteine residues in the peptide sequence is another beneficial characteristic for the production of chemically stable antimicrobials. The γ-core peptide DEFL4-20_86-110_ was the longest of all synthesized peptides; its sequence spanned the 4-Cys motif of DEFL4-20 including the γ-core. Its truncated variant DEFL4-20_92-102_ contained only the loop between the second and the third cysteines. The peptide fragment DEFL4-4_46-57_ also represented the loop between the second and the third cysteine in DEFL4-4 (Figure 1). The fragment pairs DEFL4-8_82-94_/DEFL4-8_83-93_ and DEFL4-37_90-102_/DEFL4-37_91-101_ corresponded to the above-mentioned loops in DEFL4-8 and DEFL4-37 with or without the adjacent cysteines, respectively. For comparison, a peptide fragment HvDEFL4-1_67-77_ of *Hordeum vulgare* L. HvDEFL4-1 with the same cysteine signature as the wheat 4-Cys DEFLs was also synthesized. Its sequence corresponded to the loop between the second and the third cysteines in HvDEFL4-1 (Figure 1).

#### 2.1.2. Other AMPs

The synthesized peptide fragments TkSN1_39-57_, TkThi1_96-109_, TkLTP2.25_50-62_ and TkLTPd5.6_59-75_ corresponded to the γ-cores of TkSN1, TkThi1, TkLTP2.25 and TkLTPd5.6, respectively (Figure 1), which were predicted in wheat by transcriptome sequencing (GenBank: SR7511483–SRR7511486) [20,21]. Expression profiling showed that both the selected nsLTP genes were up-regulated by the fungal infection and elicitors; furthermore, TkLTPd5.6 was highly up-regulated in IR-expressing plants [21]. The peptide fragment of Tk-AMP-K2, which was isolated from *T. kiharae* seeds and whose role remains unknown [22], encompassed its C-terminal region except for the last two residues.

In total, 20 γ-core peptides from 17 wheat AMP-like peptides and one peptide from barley were synthesized for antimicrobial activity assays.

### 2.2. Physicochemical Properties of Synthetic Peptides

The physicochemical properties of wheat and barley synthetic peptides are summarized in Table 1. The peptides contain from 11 to 25 amino acid residues. All of them are cationic. The pI (isoelectric point) values vary from 7.93 in TkThi1_96-109_ to 11.71 in DEFL4-37_91-101_. The net charge is in the range from +1 to +8. It has been generally acknowledged that the positive charge is essential for the initial electrostatic interaction of the peptide with the anionic cell surfaces of pathogenic microorganisms [24]. The aliphatic index, which reflects protein thermostability, varies from 0 in DEFL1-16_65-82_ and DEFL1-32_55-68_, since these peptides have no Ala, Val, Leu and Ile residues, to 53.08 in TkLTP2.25_50-62_ (Table 1). Thus, the structure of TkLTP2.25_50-62_ is predicted to be the most stable under a wide range of temperature regimes. The ratio of hydrophobic amino acid residues ranges from 11 in DEFL1-23_65-82_ to 55 in DEFL4-8_83-93_, DEFL4-20_92-102_ and DEFL4-37_91-101._ The average ratio of hydrophobic residues in AMPs is about 41.5%. Higher hydrophobicity facilitates the insertion of the peptide into the lipids of the microbial membranes. However, too high hydrophobicity makes the peptide insoluble in aqueous environments. The GRAVY (Grand Average of Hydropathy) index is positive for TkThi1_96-109_ and Tk-AMP-K2_10-23_ and negative for all other peptides (Table 1). Negative values mean hydrophilic proteins and positive values indicate hydrophobic proteins. The GRAVY index values range from −2 to +2. The Boman index, which is an estimate of the protein-binding potential, varies from 1.41 in DEFL4-8_82-94_ to 4.21 in DEFL1-23_65-82_. A high Boman index value (more than 2.5) indicates that an AMP can interact with a wide range of proteins, and thus, is multifunctional. Most γ-core peptides have the Boman index values exceeding 2.5. Only in six peptides are the Boman index values lower (Table 1). The hydrophobic moments (μH), which reflect the amphipathicity of an α-helix, were computed. The highest hydrophobic moment of 0.534 was in TkThi1_96-109_. Twelve peptides were predicted to be antimicrobial: all γ-core motif peptides designed from the sequences of classical DEFLs, one peptide derived from 4-Cys-containing DEFLs DEFL4-20_86-110_ that spanned the 4-Cys motif, Tk-AMP-K2_10-23_, TkLTPd5.6_59-75_ and TkSN1_39-57_.

### 2.3. 3D Structure Simulation

**Figure 2:**
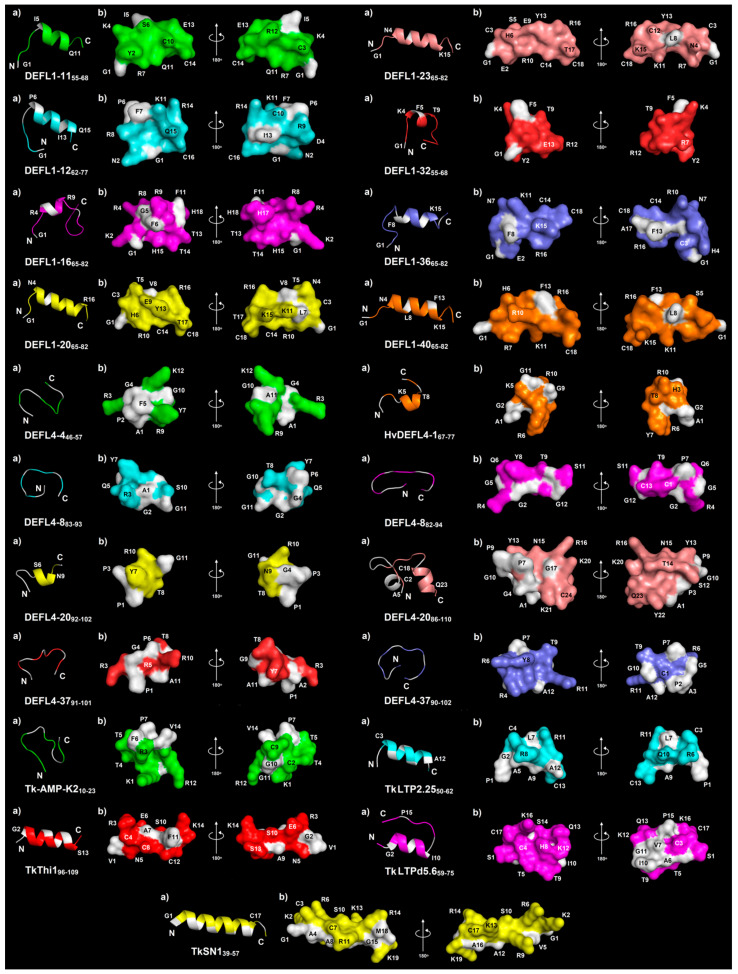
3D molecular modeling of synthetic peptides: (**a**) spatial structure (ribbon representation); (**b**) surface structure. N- and C-termini are marked with N and C, respectively. Non-polar residues are shown in white, polar residues are colored. Modeling was performed using PEP-FOLD3 [25].

The 3D structure of the γ-core peptides and their variants was predicted using PEP-FOLD3 (Figure 2) [25]. All γ-core peptides of the classical defensins possess an α-helical region of different lengths (from 6 to 13 amino acid residues) and unstructured N- and C-terminal «tails». No β-structure was predicted in either peptide. The shortest α-helices of six residues were observed in DEFL1-16_65-82_ and DEFL1-32_55-68_. In DEFL1-16_65-82_, the α-helix encompasses the conserved hexapeptide RGFRRR. In DEFL1-32_55-68_, the α-helix covers the sequence KFRRCT. The longest α-helix of 13 residues was predicted in DEFL1-20_65-82_.

The structure of the γ-core motif peptides of the 4-Cys-containing DEFLs is more diverse (Figure 2). The α-helices were predicted only in three peptides: DEFL4-20_92-102_, HvDEFL4-1_67-77_ and DEFL4-20_86-110_. The latter has two α-helical regions: in the N- and C-terminal regions of the molecule, while the central part of the peptide is unstructured. The α-helices are short and consist of four amino acid residues. In DEFL4-20_86-110_, the second α-helix is longer and contains six amino acid residues. The γ-core motif peptides from the other 4-Cys-containing DEFLs were predicted to adopt the random coil conformation.

The snakin γ-core peptide TkSN1_39-57_ adopts α-helical conformation. The α-helix encompasses most of the peptide’s length from the residue 1 to residue 17. The peptide is amphiphilic. The positively charged residues (K2, R6, K13, R14 and K19) form a distinct cluster.

Both nsLTP-derived peptides also possess a helical region, which is longer in TkLTP2.25_50-62_ (residues C3–A12) than in TkLTPd5.6_59-75_ (residues G2−I10). Two positively charged residues (R8 and R11) in TkLTP2.25_50-62_ and three positively charged residues in TkLTPd5.6_59-75_ (H8, K12 and K16) are grouped together in the 3D structure of the peptides.

In the 3D structure of TkThi1_96-109_, a helical region was also predicted (residues G2−S13). In contrast, the Tk-AMP-K2_10-23_ was predicted to be in a random coil conformation.

### 2.4. Antimicrobial Activity of Synthetic Peptides

**Figure 3:**
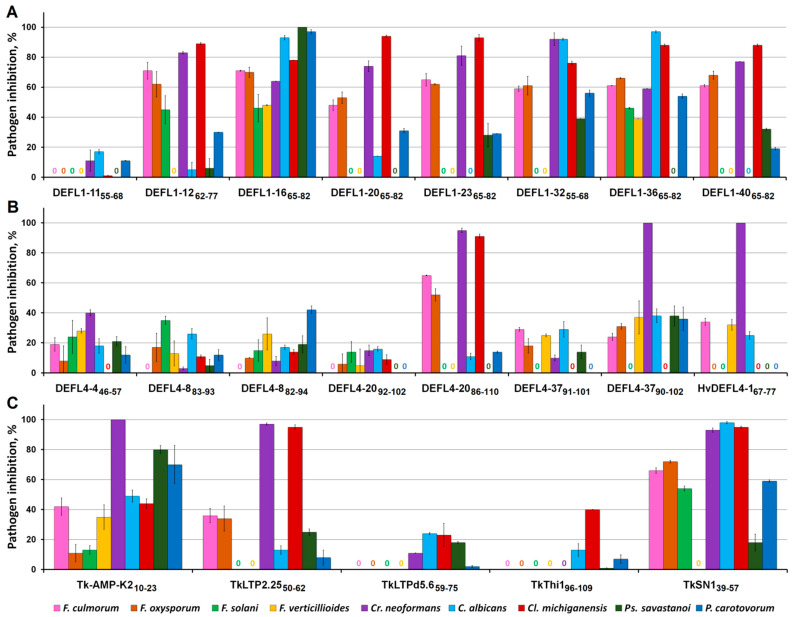
Growth inhibition of pathogens in the presence of 300 μM of synthetic peptides: (**A**) 8-Cys DEFL-derived peptides; (**B**) 4-Cys DEFL-derived peptides; (**C**) peptides derived from the knottin, nsLTPs, thionin and snakin. Bars represent the mean ± SD of growth compared to control (pathogen growth in the absence of peptide).

The antimicrobial activity of synthetic peptides was tested against a panel of human and plant pathogens. Among them were the yeasts *Candida albicans* VKM Y-2994 and *Cryptococcus neoformans* VKM Y-2755, the plant pathogenic bacteria *Clavibacter michiganensis* subsp. *michiganensis* VKM Ac-1403, *Pseudomonas savastanoi* pv. *savastanoi* VKM B-1546 and *Pectobacterium carotovorum* subsp. *carotovorum* VKM B-1247, and four *Fusarium* species (*F. oxysporum* VKM F-137, *F. culmorum* VKM F-2303, *F. solani* VKM F-142 and *F. verticillioides* VKM F-670), which cause diseases in plants and humans. The antimicrobial activity was first measured at the peptide concentration of 300 µM (Figure 3).

#### 2.4.1. γ-Cores of Classical Defensins

Most γ-cores of classical defensins displayed antimicrobial activity against the vast majority of the pathogens tested (Figure 3A). All of them, except DEFL1-11_55-68_, were highly active against *Cr. neoformans*. The inhibition varied from 64% for DEFL1-16_65-82_ to 92% for DEFL1-32_55-68_. The inhibition caused by DEFL1-12_62-77_, DEFL1-23_65-82_ and DEFL1-40_65-82_ was also very high: 83, 81 and 77%, respectively. The inhibition pattern of *C. albicans* was much more variable. While DEFL1-23_65-82_ and DEFL1-40_65-82_ failed to suppress the growth of *C. albicans* at the tested concentration, DEFL1-16_65-82_, DEFL1-32_55-68_ and DEFL1-36_65-82_ were highly efficient in suppressing its growth: inhibition amounted to 97% by DEFL1-36_65-82_, and was as high as 93 and 92% by DEFL1-16_65-82_ and DEFL1-32_55-68_, respectively. Of the pathogenic bacteria, the Gram-positive bacterium *Cl. michiganensis* was the most sensitive to the peptides (Figure 3A). The inhibition of *Cl. michiganensis* was above 76% for all peptides except DEFL1-11_55-68_ and reached 93 and 94% for DEFL1-23_65-82_ and DEFL1-20_65-82_, respectively. All the peptides inhibited *P. carotovorum*; however, the degree of inhibition was lower than for *Cl. michiganensis* and ranged from 11% for DEFL1-11_55-68_ to 97% for DEFL1-16_65-82_. Of the classical defensins’ γ-cores, DEFL1-16_65-82_ was also exceptionally effective in inhibiting *Ps. savastanoi* (100% inhibition). Conversely, DEFL1-11_55-68_, DEFL1-20_65-82_ and DEFL1-36_65-82_ failed to suppress *Ps. savastanoi*. Inhibition of *Ps. savastanoi* by other peptides ranged from 6 to 39% by DEFL1-12_62-77_ and DEFL1-32_55-68_, respectively. All the peptides except DEFL1-11_55-68_ inhibited at least two *Fusarium* species. Of the *Fusarium* fungi, *F. culmorum* and *F. oxysporum* proved much more sensitive to the γ-cores of classical defensins than *F. solani* and *F. verticillioides*. The inhibition of *F. culmorum* varied from 48% for DEFL1-20_65-82_ to 71% for DEFL1-12_62-77_ and DEFL1-16_65-82_. The inhibition of *F. oxysporum* was in the range from 53% for DEFL1-20_65-82_ to 70% for DEFL1-16_65-82_. *F. solani* was inhibited by three peptides: DEFL1-12_62-77_, DEFL1-16_65-82_ and DEFL1-36_65-82_ (45, 46 and 46% inhibition, respectively). *F. verticillioides* was suppressed only by DEFL1-16_65-82_ and DEFL1-36_65-82_ (48 and 39% inhibition, respectively).

#### 2.4.2. Peptide Fragments of 4-Cys-Containing DEFLs

Studies of the antimicrobial activity of seven peptide fragments of 4-Cys-containing DEFLs showed that all of them suppressed the growth of yeasts, however, the efficiency of inhibition varied depending on the peptide tested (Figure 3B). Usually, the activity of the longer peptides was higher than that of the truncated variants: DEFL4-20_86-110_ was more potent than DEFL4-20_92-102_, DEFL4-37_90-102_ was more efficient than DEFL4-37_91-101_, and DEFL4-8_82-94_ was more effective than DEFL4-8_83-93_ against *Cr. neoformans*. The opposite trend was observed for *C. albicans*. The peptides DEFL4-20_86-110_ and DEFL4-37_90-102_ had the highest activity against *Cr. neoformans* (95 and 100% inhibition, respectively). The efficiency of inhibition of *Cr. neoformans* by DEFL4-4_46-57_ and *C. albicans* by DEFL4-37_90-102_ was also rather high (Figure 3B). The efficiency of inhibition of yeasts by other peptide fragments was lower (equal or below 26%). Of the pathogenic bacteria, *Cl. michiganensis* was suppressed by four peptides, while *Ps. savastanoi* and *P. carotovorum* were suppressed by five. The peptide DEFL4-20_86-110_ exhibited the highest activity against *Cl. michiganensis* (91% inhibition). DEFL4-37_90-102_ showed the highest activity against *Ps. savastanoi*. *P. carotovorum* was inhibited by 42 and 36% by DEFL4-8_82-94_ and DEFL4-37_90-102_, respectively. Again, the longer peptides were more effective than the truncated derivatives. Studies of the antifungal activity of the fragments of the 4-Cys-containing DEFLs showed that *F. oxysporum* growth was suppressed by all seven peptides, *F. verticillioides* by six peptides, and *F. culmorum* and *F. solani* by four peptides. The highest activity (65% inhibition) was displayed by DEFL4-20_86-110_ against *F. culmorum*. The same peptide was the most potent against *F. oxysporum* (52% inhibition). In contrast to the activity against yeasts and bacteria, no positive correlation was observed between the length of the peptide fragment and its activity against all *Fusarium* species. While DEFL4-20_86-110_ was much more active than DEFL4-20_92-102_ against *F. culmorum* and *F. oxysporum*, the truncated peptide DEFL4-20_92-102_ was more potent against *F. solani*. The peptide DEFL4-37_90-102_ was more effective than DEFL4-37_91-101_ in inhibiting the growth of *F. oxysporum* and *F. verticillioides*; the activity of DEFL4-8_82-94_ against *F. oxysporum* and *F. solani* was lower than that of DEFL4-8_83-93_.

The barley peptide HvDEFL4-1_67-77_ showed the narrowest activity spectrum (Figure 3B). Of the nine tested pathogens, it was active against four. It showed complete inhibition of *Cr. neoformans*, while *C. albicans* was suppressed by 25%. Inhibition of *F. culmorum* and *F. verticillioides* was 34 and 32%, respectively. Other pathogens were insensitive to HvDEFL4-1_67-77_.

#### 2.4.3. Peptide Fragments of Other Wheat AMPs

The peptide fragment Tk-AMP-K2_10-23_, with a high efficiency, suppressed the growth of all the tested pathogens (Figure 3C). Similar to other peptides, its activity was pathogen-dependent. The most sensitive pathogen to the peptide was *Cr. neoformans* (100% inhibition) followed by the Gram-negative bacteria *Ps. savastanoi* (80% inhibition) and *P. carotovorum* (70% inhibition). The efficiency of inhibition of the other pathogens, such as *C. albicans*, *F. culmorum* and *F. verticillioides* was also rather high (49, 42 and 35%, respectively). The degree of inhibition of two other *Fusarium* species (*F. oxysporum* and *F. solani*) as well as the Gram-positive bacterium *Cl. michiganensis* was much lower (11, 13 and 24%, respectively).

The antimicrobial activity of the peptide fragments of wheat nsLTPs TkLTP2.25_50-62_ and TkLTPd5.6_59-75_ differed considerably: TkLTP2.25_50-62_ was much more active than TkLTPd5.6_59-75_. The peptide TkLTP2.25_50-62_ nearly completely inhibited the growth of *Cr. neoformans* and *Cl. michiganensis*. *F. culmorum* and *F. oxysporum* were inhibited by 36 and 34%, respectively. At the tested concentration, the peptide TkLTPd5.6_59-75_ was inactive against all *Fusarium* species. The activity against yeasts was 11 and 24%, and 23% against *Cl. michiganensis* (Figure 3C).

The thionin fragment TkThi1_96-109_ inhibited the growth of *Cl. michiganensis* (40% inhibition). *C. albicans* was suppressed by 13%, and *P. carotovorum* by 7%. Other pathogens were not inhibited by the peptide.

The snakin fragment TkSN1_39-57_ was a very effective inhibitor of all tested microbes except *F. verticillioides*. The efficiency of inhibition varied from 18% (*Ps. savostanoi*) to 98% (*C. albicans*). Both the yeast species and *Cl. michiganensis* were the most sensitive pathogens, which were virtually completely inhibited by TkSN1_39-57_ (Figure 3C).

### 2.5. Dynamics of Pathogen Inhibition by γ-Core Motif Peptides

**Figure 4:**
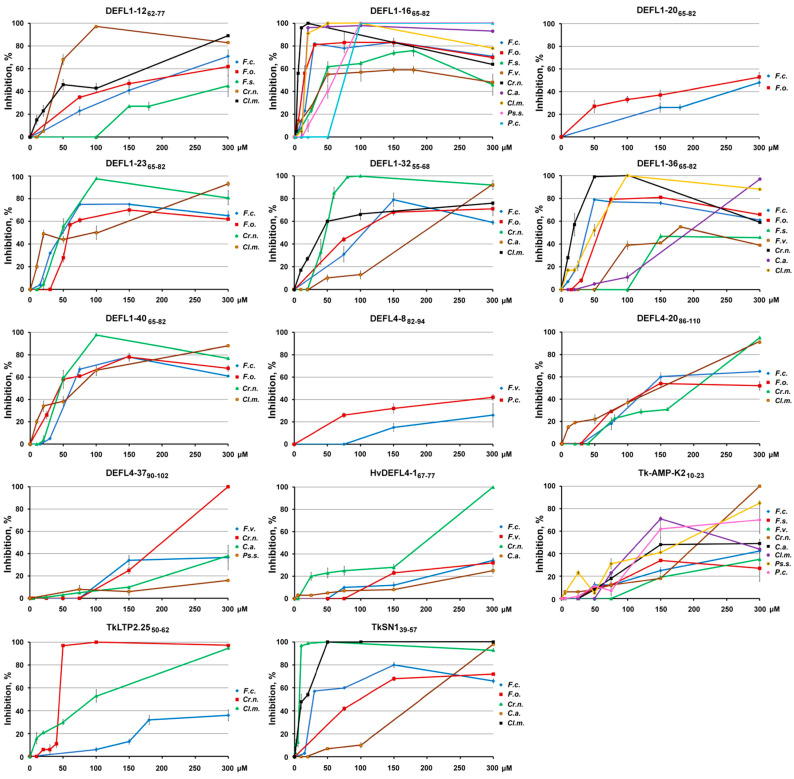
Inhibition curves of pathogenic microorganisms in the presence of different concentrations of γ-core motif peptides (relative to control, %). Error bars represent the SD of technical triplicates. The following abbreviations were used: *F.c.* for *F. culmorum*; *F.o.*, *F. oxysporum*; *F.s.*, *F. solani*; *F.v.*, *F. verticillioides*; *Cr.n.*, *Cr. neoformans*; *C.a.*, *C. albicans*; *Cl.m.*, *Cl. michiganensis*; *Ps.s., Ps. savastanoi*; and *P.c., P. carotovorum*.

The dynamics of pathogen inhibition by γ-core peptides was studied, and the IC_50_ values were calculated (Figure 4, Table 2). The efficiency of inhibition by DEFL1-12_67-77_ was the highest for *Cr. neoformans* and *Cl. michiganensis* and amounted to nearly a 100% inhibition of *Cr. neoformans* at the peptide concentration of 100 µM. The efficiency of inhibition of *Cl. michiganensis* reached 90% at the highest tested concentration of 300 µM. The peptide DEFL1-16_65-82_ was much more active against most tested pathogens, especially against *Cr. neoformans*, *C. albicans*, *Cl. michiganensis*, and two *Fusarium* species: *F. oxysporum* and *F. culmorum*. The IC_50_ values for these pathogens were in the range from 4.4 to 20.7 µM. The peptide DEFL1-20_65-82_ inhibited the growth of *F. oxysporum* and *F. culmorum*, however, inhibition did not exceed 60%. The peptide DEFL1-23_65-82_ was highly active against the yeast *Cr. neoformans* and the bacterium *Cl. michiganensis*. The inhibition of *Cr. neoformans* reached 98% at a concentration of 100 µM. *F. culmorum* and *F. oxysporum* were also highly susceptible to the peptide. The suppression of the growth of the *Fusarium* fungi amounted to 75% at the peptide concentration of 150 µM. The peptide DEFL1-32_55-68_ was highly active against five pathogens. At the peptide concentration of 80 µM, *Cr. neoformans* was inhibited by 100%. *C. albicans* was suppressed by 92% at the highest tested concentration. The inhibition efficiency of three other pathogens (*Cl. michiganensis*, *F. oxysporum* and *F. culmorum*) reached 70−79% depending on the pathogen species. The peptide DEFL1-36_65-82_ had a very broad host range, suppressing the growth of seven pathogens with the complete inhibition of *Cr. neoformans* and *Cl. michiganensis* at 50 and 100 µM, respectively. Inhibition of *F. culmorum* and *F. oxysporum* reached 80% at 50 and 70 µM, respectively. The maximum inhibition of other *Fusarium* species was in the range from 45 to 55%. The peptide DEFL1-40_65-82_ was highly active against *Cr. neoformans*, *Cl. michiganensis, F. oxysporum* and *F. culmorum*. Almost a complete inhibition of 98% for *Cr. neoformans* was achieved at the peptide concentration of 100 µM. *Cl. michiganensis* was by 88% inhibited by the highest peptide concentration tested. A maximum inhibition of 78% was achieved for the two *Fusarium* species at the peptide concentration of 150 µM.

The peptide DEFL4-8_82-94_ inhibited the growth of *F. verticillioides* and *P. carotovorum*. A maximum inhibition of 26 and 42%, respectively, was observed at the highest tested concentration. The peptide DEFL4-20_86-110_ suppressed the growth of four pathogenic microbes. *Cr. neoformans* and *Cl. michiganensis* were suppressed by 95 and 91%, respectively, at the highest peptide concentration, while *F. culmorum* and *F. oxysporum* were suppressed by 60 and 54% at 150 µM. The peptide DEFL4-37_90-102_ induced 100% inhibition of *Cr. neoformans* at the highest tested concentration. The maximum inhibition of *F. verticillioides* and *C. albicans* was about 35% at 150 and 300 µM, respectively.

The peptide HvDEFL4-1_67-77_ caused a 100% inhibition of *Cr. neoformans* growth at the highest tested concentration. The inhibition of *F. culmorum, F. verticillioides* and *C. albicans* amounted to 35, 32 and 25%, respectively.

Tk-AMP-K2_10-23_ was effective against eight pathogens. A complete inhibition of *Cr. neoformans* was reached at the highest tested concentration. Inhibition of other pathogens amounted to 85% for *Ps. savastanoi*, 70% for *Cl. michiganensis* and *P. carotovorum*, 49% for *C. albicans*, 42% for *F. culmorum*, and 34% for *F. solani* and *F. verticillioides*.

The peptide TkLTP2.25_50-62_ efficiently inhibited *Cr. neoformans* and *Cl. michiganensis*. A 100% inhibition of *Cr. neoformans* was achieved at the concentration of 100 µM, and 95% inhibition of *Cl. michiganensis* at 300 µM. Inhibition of *F. culmorum* did not exceed 36%.

The peptide TkSN1_39-57_ was a potent inhibitor of five pathogens. The most sensitive species were *Cr. neoformans* and *Cl. michiganensis*. A 100% inhibition was achieved at the peptide concentrations of 20 and 50 µM, respectively. The complete inhibition of *C. albicans* required the peptide concentration of 300 µM. An 80% inhibition of *F. culmorum* was reached with 150 µM of TkSN1_39-57_. The maximum inhibition of *F. oxysporum* (68%) was achieved at the peptide concentration of 150 µM.

### 2.6. Staining with Propidium Iodide

**Figure 5:**
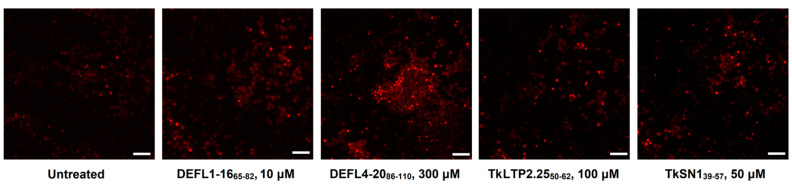
Fluorescence microscopy images of *Cr. neoformans* cells incubated in the presence of peptides and stained with propidium iodide. Untreated *Cr. neoformans* cells were used as a negative control. Scale bar = 20 μm.

**Figure 6:**
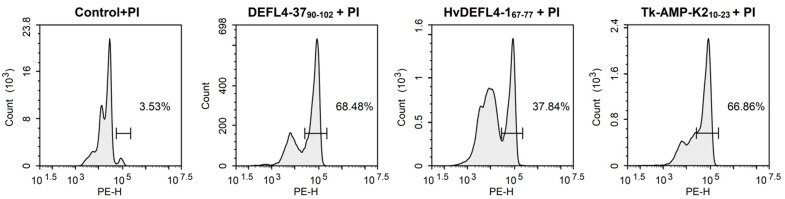
Penetration of propidium iodide (PI) into *Cr. neoformans* cells treated with peptides at a concentration of 300 µm. Untreated *Cr. neoformans* cells were used as a negative control.

**Figure 7:**
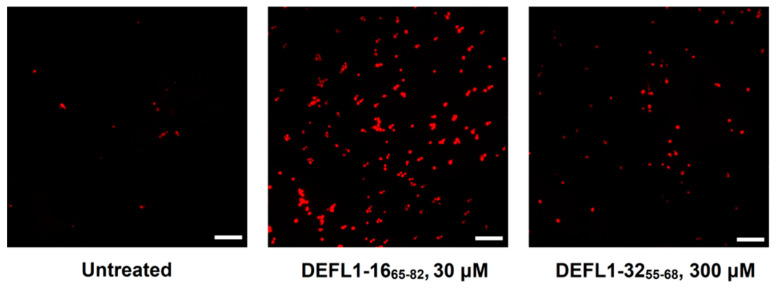
Fluorescence microscopy images of *C. albicans* cells incubated in the presence of peptides and stained with propidium iodide. Untreated *C. albicans* cells were used as a negative control. Scale bar = 20 μm.

To get an insight into the mode of action of the most active synthetic peptides, eight γ-core peptides were chosen: DEFL1-16_65-82_, DEFL1-32_55-68_, DEFL4-20_86-110_, DEFL 4-37_90-102_, HvDEFL4-1_67-77_, TkLTP2.25_50-62_, TkSN1_39-57_ and Tk-AMP-K2_10-23_. Their ability to disturb the integrity of pathogen membranes was assayed by staining with propidium iodide of *C. albicans* and *Cr. neoformans* cells preliminarily incubated with the peptides. The results were visualized by fluorescent microscopy and quantified by flow cytometry (Figure 5, Figure 6 and Figure 7). All tested peptides induced membrane permeabilization resulting in the accumulation of the fluorescent dye inside the yeast cells.

## 3. Discussion

The survival of the growing human population worldwide depends on agricultural production. Among phytopathogens, fungi are the key causative agents of destructive crop plant epidemics; furthermore, they cause considerable and persistent losses of crop yields annually [26]. Plant diseases caused by fungi alone usually reduce by as much as a 30 percent of the crop harvest in the absence of appropriate and efficient management [27]. Bacterial diseases, which are widespread on all kinds of cultivated and commercial value plants and are especially difficult to identify and control, are also of great economic concern. The control of fungal and bacterial diseases is mainly based on the application of chemical fungicides. Only few pesticides including antibiotics and heavy metals are available to control bacterial infections. Chemical fungicides inhibit pathogens but are toxic to beneficial microorganisms, human and animal health, and the environment. Moreover, the emergence of resistant strains makes plant diseases increasingly challenging to treat. In addition to reduced agricultural food production, fungi can infect and cause diseases in humans, which affect approximately 25% of the total population and increase the rate of morbidity and mortality among immunocompromised individuals [28].

Accordingly, the development of ecologically friendly and non-toxic strategies to control fungal and bacterial infections of plants and opportunistic fungal infections in humans has become a priority. These alternative control measures include the use of plant-derived natural antimicrobials and biological control agents.

The effective use in disease control of plant AMPs as natural antibiotics requires the knowledge of their antimicrobial determinants and mode of action. The efficient exploitation of the biocontrol agents in plant disease management is also dependent on the elucidation of their mechanism of action. Earlier, we demonstrated that the intracellular metabolites of the non-pathogenic strain FS-94 of *F. sambucinum* induced resistance to the pathogenic *F. oxysporum* strains F37 and F-137 in tomato and wheat plants, respectively. Resistance development was accompanied by the up-regulation of an array of AMP-like genes [19,21,29]. However, their exact biological role in the immune response remained unknown. Structure–function studies of the classical defensins showed that the antimicrobial activity is associated, at least in part, with their γ-core motifs [17,30]. In our studies of tomato CRPs, we showed that the γ-core motifs of the CRPs belonging to other peptide families, such as snakins, MEG (Maternally Expressed Gene) peptides, and nsLTPs, also possess antimicrobial activity [18]. However, it remained unclear as to whether the γ-cores of the so-called 4-Cys DEFLs, knottin- and thionin-like peptides display antimicrobial properties. In this work, we assayed the antimicrobial activity of the γ-core peptides from the wheat AMPs belonging to the classical defensins, 4-Cys DEFLs, snakins, nsLTPs, knottins and thionins, which were up-regulated by the pathogenic fungus *F. oxysporum* or *F. sambucinum* resistance inducers, against an array of human and plant pathogens. We showed that all the tested γ-core peptides exhibited antimicrobial properties. This important finding proves the role of this motif in antimicrobial properties of AMPs of different families. It also assigns antimicrobial functions to the peptides, whose role in defense has not been elucidated so far.

### 3.1. Discovery of Antimicrobial Activity in 4-Cys DEFLs and Conservation of the γ-Core Motifs

For the first time, we detected antimicrobial activity in the γ-core peptides of several 4-Cys-containing DEFLs which proves their direct role in defense as antimicrobial agents. Note that until now, only two families of 4-Cys-containing AMPs have been reported in plants—α-hairpinins and NCR (Nodule Cysteine-Rich) peptides. In the hairpinins, discovered in a number of species including maize, wheat, chickweed, buckwheat, macadamia and some others, the cysteine arrangement is as follows: C-X{3}-C-X{n}-C-X{3}-C [31]. The fold of the hairpinins resembles a hairpin composed of two α-helices connected by two disulphide bridges. Hairpinins exert either antifungal or proteinase inhibitory activity [31]. NCR peptides comprise a large family of highly diverse peptides with a conserved 4- or 6-Cys motif which are specifically expressed in legume nodules [32]. The cysteine motif in 4-Cys-containing NCR peptides conforms to the following signature: C-X-{5}-C-X{n}-C-X{4}-C. Their 3D structure includes either one short C-terminal antiparallel β-sheet and a short α-helix as in NCR044 or only the β-sheet without α-helix as in NCR169 [33,34]. NCR peptides were shown to be involved in nodule development and defense [35].

The cysteine signature in 4-Cys DEFLs of wheat and barley is different from those of α-hairpinins and NCR peptides, and their 3D structure as well as functions remains obscure. Three of the wheat DEFLs taken for the γ-core peptide synthesis showed a high sequence similarity to the uncharacterized proteins of wheat diploid relatives, and one DEFL displayed no significant homology to any of the protein sequences in the public databases (Figures S1, S2 and S4). Interestingly, homologues of the studied wheat 4-Cys DEFLs differing in the degree of sequence similarity/identity were discovered in other cereals (Figures S1–S5). For example, DEFL4-8_82-94_ of the hexaploid *T. kiharae* showed high sequence similarity and identity in the γ-core region with the predicted 4-Cys DEFLs from the closely related species, such as *Ae. tauschii*, *Triticum turgidum* L. and *Triticum aestivum* L. High sequence similarity, especially in the γ-core motif region, was observed in the predicted 4-Cys DEFLs from more distantly related species, such as *Thinopyrum elongatum* (Host) D. R. Dewey (syn. *Elytrigia elongata*), *Oryza* sp., *Zizania palustris* L., *Eragrostis curvula* (Schrad.) Nees, and *Brachypodium distachyon* (L.) P. Beauv. (Figure S2). The γ-core motif identical to DEFL4-20_86-110_ was discovered in DEFL4-34, DEFL4-35 and *T. elongatum* TeDEFL4-20 (Figure S3, Table S1). Closely related sequences were found in 4-Cys DEFLs from the wheat DEFL4-10, KQJ82122.1 from *B. distachyon* and XP_002447609.1 from *Sorghum bicolor* (L.) Moench. DEFL4-37_90-102_ was discovered in KAE8796982.1 from *H. vulgare*, with minor modifications, in the predicted *T. elongatum* 4-Cys DEFL, KAF7033286.1 of *T. aestium*, XP_037418557.1 from *Triticum durum* Desf., XP_020190562.1 from *Ae. tauschii* and DEFL4-38 from *T. kiharae* (Figure S4). Sequences related to DEFL4-4_46-57_ were found in predicted DEFLs of several *Triticum* and *Aegilops* species, TeDEFL4-4 from *T. elongatum*, KQJ82122.1 from *B. distachyon* and XP_044973437.1 from *H. vulgare* (Figure S1). The widespread distribution of 4-Cys DEFLs among cereals and the conservation of their γ-core sequences in evolution point to the important role of this peptide family in the plant immune system and the significance of the γ-core motif for the antimicrobial activity. It is worth noting that conservation of the γ-core sequences was observed not only in 4-Cys DEFLs, but in other AMP families: classical defensins, snakins, nsLTPs, thionins (Figures S6–S17), in which family members with identical γ-core motifs were discovered. For example, the γ-core sequence of DEFL1-16 shown to exert potent antimicrobial properties was detected in DEFLs from different species of grasses including both cultivated and wild-growing (Figure S8). Furthermore, this sequence was found in the defensins of plants beyond the grass family [23].

### 3.2. Discovery of Antimicrobial Activity in the Knottin-like Peptide Tk-AMP-K2_10-23_ and the C-Terminal Prodomain of the Thionin-like Protein TkThi1_96-109_

In addition to 4-Cys DEFLs, we revealed antimicrobial activity in the knottin-like peptide Tk-AMP-K2_10-23_, which was earlier isolated from *T. kiharae* seeds [22]. According to the cysteine signature similar to that in conotoxins, this peptide was assigned to knottin-like peptides. In this work, we confirmed the antimicrobial activity of this peptide.

We also assayed the antimicrobial potential of the γ-core motif of the thionin-like peptide TkThi1. This motif was discovered not in the mature peptide domain, but in the prodomain of the TkThi1 preproprotein. Note, that all thionins are synthesized as preproproteins that contain a signal peptide and an acidic C-terminal propeptide [36]. Within species, the C-terminal propeptides, especially the position of the six cysteine residues in the polypeptide chain, are highly conserved [37]. There is also homology, although less pronounced, between the acidic propeptides of the thionins of different plant species. We discovered sequence similarity between the γ-core motifs of the prodomains of the wheat thionin-like peptide TkThi1 and the *Oryza sativa* L. thionin-like proteins (EAZ01134.1, XP_015641946.1, etc.) (Figure S16). The sequence conservatism of the C-terminal prodomain indicates the functional importance of this precursor region, although its exact role has not been clarified so far. The C-terminal prodomain is thought to be essential for the targeted transport of the mature thionin to the vacuoles, cell walls or protein bodies. In addition, it is believed to neutralize the toxic properties of the mature peptide before it enters the intercellular space or the vacuole or acts as a chaperone that ensures thionin folding [38]. Our results show that TkThi1_96-109_ exhibits antimicrobial activity, with the highest activity against the Gram-positive bacterium *Cl. michiganensis* (40% inhibition at the highest tested concentration). Therefore, previously unknown antimicrobial functions of the C-terminal thionin prodomain have been disclosed.

### 3.3. Antimicrobial Potency and Activity Spectrum of the γ-Core Peptides

In addition to revealing antimicrobial properties in CRPs with previously unknown functions, we also compared the antimicrobial potency and activity spectrum of all synthetic γ-core peptides to infer the problem of structure–function relationships and identify the peptides most suitable for control of particular pathogens.

Antimicrobial assays of synthetic γ-core peptides revealed that all of them were active against several pathogens. However, the degree of inhibition as well as the activity spectrum varied depending on the peptide and the pathogen species. This might result from the different physicochemical characteristics of the tested synthetic peptides.

All except one peptide (TkThi1_96-109_) suppressed the growth of the opportunistic human pathogen *Cr. neoformans* (Figure 3). Several peptides (DEFL4-37_90-102_, HvDEFL4-1_67-77_ and Tk-AMP-K2_10-23_) were exceptionally active and completely inhibited the growth of this pathogen. The efficiency of inhibition of *Cr. neoformans* by several other γ-core peptides (DEFL1-32_55-68_, DEFL4-20_86-110_, TkSN1_39-57_ and TkLTP2.25_50-62_) was also very high (above 92%). Notably, the peptide DEFL4-37_91-101_, which is identical to DEFL4-37_90-102_, but lacks the N- and C-terminal cysteine residues, is 10 times less active than DEFL4-37_90-102_, pointing to the importance of the terminal cysteines in this peptide for the activity against *Cr. neoformans* (Figure 3). The same holds true for the peptide pair DEFL4-20_86-110_ and the truncated variant DEFL4-20_92-102_ (95 versus 15% inhibition). At the same time, the peptide HvDEFL4-1_67-77_ from *H. vulgare* HvDEFL4-1, which also has no terminal cysteine residues as seen in the wheat DEFL4-37_91-101_, is highly active against this pathogen (Figure 3). Since the net charge of all the three compared peptides (DEFL4-37_91-101_, DEFL4-37_90-102_ and HvDEFL4-1_67-77_) is the same (+3), the amino acid sequence of HvDEFL4-1_67-77_ might account for its high inhibitory activity. Several DEFL1 γ-core peptides were also potent inhibitors of *Cr. neoformans*, with the degree of inhibition ranging from 59% for DEFL1-36_65-82_ to 83% for DEFL1-12_62-77_.

*C. albicans* was effectively suppressed by four tested peptides: DEFL1-16_65-82_, DEFL1-32_55-68_, DEFL1-36_65-82_ and TkSN1_39-57_ (Figure 3). The efficiency of inhibition was above 92%. Thus, two peptides, DEFL1-32_55-68_ and TkSN1_39-57_, were equally highly active against both tested yeast species.

Of the plant pathogenic bacteria, the peptides tested were much more active against the Gram-positive bacterium *Cl. michiganensis* than against the Gram-negative bacteria *Ps. savastanoi* and *P. carotovorum* (Figure 3). Ten peptides showed high activity against *Cl. michiganensis*. The most potent inhibitors of *Cl. michiganensis* were TkLTP2.25_50-62_ and TkSN1_39-57_ with the degree of inhibition of 95%, and DEFL1-20_65-82_, DEFL1-23_65-82_ and DEFL4-20_86-110_ with the degree of inhibition of 94, 93, and 91%, respectively. Other DEFL1-derived γ-core peptides (DEFL1-12_62-77_, DEFL1-16_65-82_, DEFL1-32_55-68_, DEFL1-36_65-82_ and DEFL1-40_65-82_) were also highly active against this pathogen (the degree of inhibition above 76%) (Figure 3). The longer variant DEFL4-20_86-110_ was much more active than its truncated variant DEFL4-20_92-102_ (91 versus 9% inhibition).

*Ps. savostanoi* was efficiently inhibited by two peptides: DEFL1-16_65-82_ (100% inhibition) and Tk-AMP-K2_10-23_ (80% inhibition) (Figure 3). The same two peptides displayed the greatest inhibitory activity against the second tested Gram-negative bacterium *P. carotovorum*. The degree of inhibition was 97% for DEFL1-16_65-82_ and 70% for Tk-AMP-K2_10-23_. For three other peptides DEFL1-32_55-68_, DEFL1-36_65-82_, TkSN1_39-57_ the degree of *P. carotovorum* inhibition was above 50% (56, 54 and 59%, respectively). The moderately active peptide DEFL4-37_90-102_ was more active than its truncated version DEFL4-37_91-101_ (36 versus 0% inhibition).

The antifungal assays with the *Fusarium* species showed that *F. culmorum* and *F. oxysporum* were much more sensitive to the γ-core peptides than *F. solani* and *F. verticillioides* (Figure 3). However, complete inhibition of fungal growth was not achieved with either peptide. Eight peptides (all DEFL1-derived peptides except DEFL1-11_55-68_, DEFL4-20_86-110_ and TkSN1_39-57_) displayed moderate activity against *F. culmorum* (the degree of inhibition being around 50%). The same peptides showed the greatest activity against *F. oxysporum*. The longer peptide DEFL4-20_86-110_ was much more active than the truncated variant DEFL4-20_92-102_ against *F. culmorum* and *F. oxysporum*. The maximum inhibitory activity (54%) against *F. solani* was observed with the TkSN1_39-57_ peptide. Three other DEFL1-derived γ-core peptides (DEFL1-12_62-77_, DEFL1-16_65-82_ and DEFL1-36_65-82_) also showed good inhibitory activity (45, 46 and 46% inhibition, respectively). A 48% inhibition of *F. verticillioides* was achieved with DEFL1-16_65-82_. Other peptides showed moderate or weak activity.

The activity spectrum was the broadest for DEFL1-16_65-82_ and Tk-AMP-K2_10-23_: both peptides inhibited all nine tested pathogens (Figure 3). However, the degree of inhibition of most pathogens was higher for DEFL1-16_65-82_. Six peptides were active against eight of nine pathogens. Of them, DEFL1-36_65-82_ and TkSN1_39-57_ had the highest activity against the sensitive microorganisms. The high inhibitory activity of DEFL1-16_65-82_, DEFL1-36_65-82_ and TkSN1_39-57_ against most tested pathogens correlates with the high net positive charge of their molecules (+8 and +7, +5, respectively). Conversely, the low charge of the molecules (+1) correlates with the low antimicrobial potency of DEFL4-8_83-93_, DEFL4-8_82-94_, DEFL4-20_92-102_ and TkThi1_96-109_. The importance of the high charge for the antimicrobial activity of α-helical peptides was shown by Zelezetsky and Tossi [39]. On the model peptides it was demonstrated that decreasing the charge below +3 reduced antimicrobial potency [39]. It was also shown that the activity depended on the overall cationicity and not necessarily on the location of positively charged residues in the polypeptide chain [39]. On the whole, the tested γ-core peptides with the net charge of +1 were less active than those with the higher net charge. However, besides the charge, other factors also contributed to the antimicrobial activity of the γ-core peptides. This follows from the observation that the peptides with an identical charge differed in antimicrobial properties. The importance of the amphiphilic structure for potent, broad-spectrum activity was demonstrated for the α-helical AMPs [39]. However, our experiments show that the peptide TkThi1_96-109_ with the highest hydrophobic moment of 0.534 and predicted to be α-helical exhibited moderate and narrow-range activity when compared to other peptides. The role of hydrophobicity in the antimicrobial activity of α-helical peptides was also repeatedly emphasized. Hydrophobicity is assumed to be significant for penetration of the peptide into the lipid bilayer of the membranes of pathogens. In our assays, the ratio of hydrophobic residues in one of the most potent peptides DEFL1-16_65-82_ as well as its hydrophobic moment was rather low when compared to much more hydrophobic and amphiphilic but less-active peptides, such as TkThi1_96-109_ and Tk-AMP-K2_10-23_. In summary, our results show that any, even subtle changes in the physicochemical properties of peptides can have a pronounced effect on the potency and spectrum of antimicrobial activity.

### 3.4. Structure–Function Relationships

The comparison of antimicrobial properties of the γ-core motif peptides with high sequence similarity allowed us to draw conclusions on the role of certain amino acid residues in the antimicrobial activity. Thus, by analyzing the antimicrobial activity of DEFL-derived peptides, we discovered that DEFL1-32_55-68_ was among the most potent peptides. Conversely, DEFL1-11_55-68_ was among the least active peptides. However, DEFL1-11_55-68_ differs from DEFL1-32_55-68_ only by two amino acid residues: a dipeptide IS in DEFL1-11_55-68_ is substituted by FR in DEFL1-32_55-68_. This substitution increases the charge of DEFL1-32 (Table 1), affects its 3D structure and dramatically enhances its antimicrobial potency (Figure 3 and Figure 4). Accordingly, the dipeptide FR in the γ-core sequence of DEFL1-32 is vital for its antimicrobial activity.

A comparison of the antimicrobial properties of a pair DEFL1-23_65-82_/DEFL1-40_65-82_, which differ by a single aromatic residue (Y13F) between the second and the third cysteines, shows that the activity spectrum of both peptides is very similar, as well as the antimicrobial potency against the tested microbes, pointing to the minor role of Y versus F in the antimicrobial activity of these peptides. Sequence variation in another pair, DEFL1-20_65-82_/DEFL1-23_65-82_, which consists of a single non-conserved substitution of L7 in DEFL1-20_65-82_ for R7 in DEFL1-23_65-82_ resulting in a higher positive charge of DEFL1-23_65-82_, had a more pronounced effect on the degree of pathogen inhibition: DEFL1-20_65-82_ was unable to suppress the growth of *Ps. savastanoi*, while DEFL1-23_65-82_ inhibited this pathogen by 30% (Figure 3). DEFL1-23_65-82_ was also more active than DEFL1-20_65-82_ against *F. culmorum*, *F. oxysporum*, and *Cr. neoformans* indicating the importance of R7 in the γ-core motif for the antimicrobial activity against these pathogens. At the same time, DEFL1-20_65-82_ inhibited *C. albicans* by 14%, while DEFL1-23_65-82_ was ineffective against this pathogen.

Our results show that the charge of the peptide is not the only determinant of antimicrobial activity. This follows from the comparison of the antimicrobial properties of the pair DEFL1-20_65-82_/DEFL1-32_55-68_. The peptides have the same net charge; however, their sequences vary, which correlates with differences in antimicrobial activity against particular pathogens. In contrast to DEFL1-20_65-82_, DEFL1-32_55-68_ is active against *Ps. savastanoi* and highly active against *Cr. neoformans* and *C. albicans*, while DEFL1-20_65-82_ shows better activity against *Cl. michiganensis*. This result shows that besides the charge of the peptide, its amino acid sequence is essential for the antimicrobial activity. The same holds true for the pair TkLTP2.25_50-62_/TkLTPd5.6_59-75_. Both TkLTP2.25_50-62_ and TkLTPd5.6_59-75_ have the same net charge of +3 at pH 7.0. However, the antimicrobial activity of both peptides differs considerably: TkLTP2.25_50-62_ was much more active against the majority of tested pathogens. This means that the sequence of the nsLTP-derived peptides is likely to influence the antimicrobial activity despite the overall similarity in the 3D structure.

To elucidate the role of the terminal cysteine residues in the antimicrobial activity of 4-Cys DEFLs, we compared the peptide pairs DEFL4-8_83-93_/DEFL4-8_82-94_, DEFL4-20_92-102_/DEFL4-20_86-110_ and DEFL4-37_91-101_/DEFL4-37_90-102_. For the pair DEFL4-8_83-93_/DEFL4-8_82-94_, the addition of cysteines to the N- and C-termini increased activity against all bacterial pathogens. Conversely, against *Fusarium* species, especially *F. solani*, as well as the yeast *C. albicans*, the activity was higher for the shorter peptide DEFL4-8_83-93_. For the pair DEFL4-37_91-101_/DEFL4-37_90-102_, the terminal cysteines increased the activity against the vast majority of the pathogens except for *F. culmorum*. In the pair DEFL4-20_92-102_/DEFL4-20_86-110_, in which DEFL4-20_86-110_ is much longer than DEFL4-20_92-102_ and its charge is higher, the activity against the Gram-positive *Cl. michiganensis* and the yeast *Cr. neoformans*, as well as against two *Fusarium* species (*F. oxysporum* and *F. culmorum*) was dramatically higher for the longer and more cationic peptide DEFL4-20_86-110_. Conversely, *C. albicans* was more sensitive to the shorter peptide DEFL4-20_92-102_. In conclusion, the activity of AMP-derived γ-core peptides is not determined by a single factor but by a combination of different factors including the charge, sequence, hydrophobicity and others.

### 3.5. Mode of Action

Studies of the mode of action of the eight most active γ-core motif peptides from the AMPs of different families (DEFLs, snakins, nsLTPs, knottins) on yeast cells using staining with propidium iodide showed that all the tested peptides induced membrane permeabilization (Figure 5, Figure 6 and Figure 7). For three peptides (DEFL4-37_90-102_, HvDEFL4-1_67-77_ and Tk-AMP-K2_10-23_), flow cytometry showed a concomitant reduction in the number of *Cr. neoformans* cells, pointing to the peptide-induced cell lysis. Accordingly, the tested peptides disturbed the membrane integrity, which could be the cause of the pathogen growth arrest and cell lysis, either alone or in combination with some other mechanism, e.g., inhibition of DNA or protein synthesis, etc. We can hypothesize that the cationic nature of the peptides provided an initial interaction with the negatively charged phospholipids of the plasma membranes, followed by the formation of pores or membrane disruption by some other mechanism resulting in leakage of the cell constituents. Inhibition dynamics studies of DEFL1-32_55-68_ against *C. albicans* and DEFL4-37_90-102,_ HvDEFL4-1_67-77_ and Tk-AMP-K2_10-23_ against *Cr. neoformans* demonstrated a low inhibition level until a certain critical peptide concentration was achieved, resulting in near-complete inhibition of the pathogen (Figure 4). We speculate that such inhibition dynamics are consistent with the carpet model of membrane disintegration by the membrane-active peptides. In the carpet model, the peptides accumulate in parallel on the surface of the membrane. At a threshold concentration of peptides, the displacement of the phospholipids occurs with subsequent membrane disruption [40].

## 4. Materials and Methods

### 4.1. Chemical Synthesis of Peptide Fragments Derived from AMP-like Peptides

Peptide fragments of wheat and barley AMP-like peptides were produced by solid-phase synthesis using Fmoc chemistry (Elabscience Biotechnology Inc., Wuhan, China). The synthesized peptides were purified by RP-HPLC. Their identity to the required sequences was proved by matrix-assisted laser desorption/ionization time-of-flight (MALDI-TOF) mass spectrometric analysis on an Ultraflex MALDI-TOF mass spectrometer (Bruker Daltonics, Bremen, Germany) in a linear or reflector positive ion mode using α-cyano-4-hydroxycinnamic acid as a matrix.

The following characteristics of the synthesized peptides were calculated using the ExPASy ProtParam tool [41]: molecular weight, pI, net charge at pH 7, GRAVY index and aliphatic index. Hydrophobic moment μH was calculated with HeliQuest [42]. The Boman index was computed using APD3 [43]. The prediction of antimicrobial properties was carried out with CAMPR3 [44].

### 4.2. 3D Structure Modeling

The spatial structure of the synthesized peptides was de novo modeled using the PEP-FOLD3 program [25]. The best representative models were those with the lowest sOPEP values provided by PEP-FOLD3.

### 4.3. Antimicrobial Assays

The antimicrobial activity of synthetic peptides was tested against the yeasts *Candida albicans* VKM Y-2994 and *Cryptococcus neoformans* VKM Y-2755, the bacteria *Pseudomonas savastanoi* pv. *savastanoi* VKM B-1546, *Pectobacterium carotovorum* subsp. *carotovorum* VKM B-1247 and *Clavibacter michiganensis* subsp. *michiganensis* VKM Ac-1403, and four *Fusarium* species: *F. culmorum* VKM F-2303, *F. oxysporum* VKM F-137, *F. solani* VKM F-142 and *F. verticillioides* VKM F-670. All cultures were obtained from the All-Russian Collection of Microorganisms (VKM). The details of the production and storage of cultures of microorganisms used in this study are described at the VKM Home page [45]. Antimicrobial assays were accomplished according to the procedure described earlier [18]. In short, yeasts were grown on the YPD-P medium and the bacteria *P. carotovorum* and *Cl. michiganensis* were cultured on a modified YPD-P medium [18]. The *Fusarium* fungi were grown on potato dextrose agar at 25 °C for 7–8 days, and spores were washed off from the surface of the mycelia with sterile, distilled water. The antimicrobial activity of the peptides was measured in immunoassay microtiter plates according to the protocol of Broekaert et al. [46]. With yeasts and bacteria, each well contained 10 µL of the tested peptide solution (final concentrations of 10–300 µM) in water, 80 µL of the medium and 10 µL of the microbial suspension. The plates were incubated at 30 °C for 24 h, and the absorbance of the suspension was measured on an Efos 9305 spectrophotometer (Sapphire, Moscow, Russia) at 594 nm. For *Fusarium* species, the wells of a microtiter plate were filled with 90 µL of the fungal spore suspension in half-strength potato dextrose broth at a concentration of 2000–3000 spores in 100 µL and 10 µL of aqueous solutions of peptides at final concentrations of 10–300 µM. After 38 h of incubation, the absorbance was recorded at 595 nm on a FilterMax F5 Multi-Mode Microplate Reader (Molecular Devices, San Jose, CA, USA). Antimicrobial activity is expressed in IC_50_ values, which show the concentration necessary for 50% inhibition of the pathogen growth; this was determined from a graph showing the activity dependence on peptide concentration.

### 4.4. Statistical Analysis

For each pathogen, experiments on the peptide antimicrobial activity evaluation were carried out in triplicate per treatment. Mean values, standard deviations (SD), and the significance of differences (*p* ≤ 0.05) of the means between treatments and controls (*t*-test for independent variables) were determined using STATISTICA v. 6.1 software (StatSoft Inc., Tulsa, OK, USA).

### 4.5. Staining of Yeast Cells with Propidium Iodide

Staining of yeast cells with propidium iodide in the presence of the γ-core peptides was carried out according to the protocol described earlier [18]. *C. albicans* or *Cr. neoformans* cells (24 h culture in YPD-P at a cell concentration of 2 × 10^8^/mL) were incubated with the peptide at a concentration of 300 µM at 30 °C for 1 h. After incubation, 1 mL of the cells was stained with 0.03 mM propidium iodide (Sigma, St. Louis, MO, USA). The staining proceeded for 15 min at 30 °C. The fluorescence was recorded on an AXIO Imager A1 fluorescence microscope (ZEISS, Göttingen, Germany) using a Zeiss filter set 56 HE.

For flow cytometry experiments with *Cr. neoformans*, the yeasts were grown on the YPD medium at 28 °C for 48 h. After that, the cells were centrifuged at 12,000× *g* for 3 min, washed with MiliQ water and incubated with the peptides dissolved in water at a concentration of 300 µM at 28 °C for 2 h. The cells were stained with propidium iodide by direct addition of the dye to the incubation medium (final concentration of 2 µg/mL) and measured on a NovoCyte Flow cytometer (Agilent, Santa Clara, CA, USA) using the channel FL2 (488 nm excitation and 585 nm emission). The sample without peptides was used as a control.

## 5. Conclusions

In this work, we report the antimicrobial activity of 21 γ-core peptides of 18 AMPs of wheat *T. kiharae* and barley *H. vulgare* against an array of plant and human pathogens. All the tested peptides displayed antimicrobial properties which depended on the pathogen species. Conservation of the γ-core motif regions in AMPs of the grass species was revealed. Peptides especially efficient against particular pathogens, which may find practical application for design of novel antimicrobials, were revealed. The discovery of antimicrobial activity in 4-Cys DEFLs, the knottin-like peptide Tk-AMP-K2 and the C-terminal prodomain of the thionin-like protein TkThi1 points to their direct role in defense mechanisms. Studies of the mode of action of the eight most potent peptides against yeast cells show that they trigger membrane permeabilization resulting in cell lysis. Whether the peptides interact with intracellular targets is still to be explored.

## Figures and Tables

**Table 1:** Physicochemical properties of AMP-based synthetic peptides.

Peptide	Length, aa	Molecular Weight, Da	Net Charge at pH 7	pI	GRAVY Index	μH	Aliphatic Index	Boman Index	Ratio of Hydrophobic Residues, %	AMP Prediction
DEFL1-11_55-68_	14	1649.95	+2	8.53	−0.614	0.177	27.86	3.06	14	AMP
DEFL1-12_62-77_	16	1856.20	+3	8.98	−0.812	0.317	24.38	3.51	31	AMP
DEFL1-16_65-82_	18	2205.59	+8	9.89	−1.006	0.180	0	3.96	22	AMP
DEFL1-20_65-82_	18	2143.51	+3	8.52	−0.867	0.227	37.78	3.11	17	AMP
DEFL1-23_65-82_	18	2186.53	+4	8.92	−1.356	0.086	21.67	4.21	11	AMP
DEFL1-32_55-68_	14	1753.07	+3	8.94	−1.000	0.109	0	4.02	14	AMP
DEFL1-36_65-82_	18	2111.47	+5	8.94	−0.672	0.047	11.11	2.81	28	AMP
DEFL1-40_65-82_	18	2170.53	+4	8.94	−1.128	0.080	21.67	4.04	17	AMP
DEFL4-4_46-57_	12	1310.48	+3	11.00	−0.975	0.109	16.67	2.75	50	Non-AMP
DEFL4-8_83-93_	11	1050.10	+1	8.79	−1.109	0.223	7.69	1.9	55	Non-AMP
DEFL4-8_82-94_	13	1256.37	+1	8.06	−0.554	0.039	7.69	1.41	40	Non-AMP
DEFL4-20_92-102_	11	1076.13	+1	9.18	−1.218	0.151	9.09	2.09	55	Non-AMP
DEFL4-20_86-110_	25	2616.99	+4	9.21	−0.896	0.148	12.00	2.02	46	AMP
DEFL4-37_91-101_	11	1201.35	+3	11.71	−1.445	0.130	18.18	3.81	55	Non-AMP
DEFL4-37_90-102_	13	1407.63	+3	9.69	−0.838	0.123	15.38	3.03	46	Non-AMP
HvDEFL4-1_67-77_	11	1159.27	+4	11.00	−1.627	0.222	9.09	3.38	45	Non-AMP
Tk-AMP-K2_10-23_	14	1530.86	+3	9.98	0.021	0.315	20.71	1.89	36	AMP
TkLTP2.25_50-62_	13	1404.70	+3	9.36	−0.177	0.102	53.08	2.7	38	Non-AMP
TkLTPd5.6_59-75_	17	1720.01	+3	8.66	−0.112	0.372	45.88	0.98	35	AMP
TkThi1_96-109_	14	1474.69	+1	7.93	0.050	0.534	35.00	1.8	36	Non-AMP
TkSN1_39-57_	19	2052.53	+7	10.96	−0.553	0.198	36.32	3.17	42	AMP

**Table 2:** Antimicrobial activity of γ-core motif peptides.

Peptide	IC_50_, μM								
	*F. culmorum*	*F. oxysporum*	*F. solani*	*F. verticillioides*	*Cr. neoformans*	*C. albicans*	*Cl. michiganensis*	*Ps. savastanoi*	*P. carotovorum*
DEFL1-12_62-77_	>100	>100	>100	−	37.3 ± 1.6	−	69.1 ± 1.2	−	−
DEFL1-16_65-82_	20.7 ± 1.7	12.1 ± 2.4	52.5 ± 6.2	48.4 ± 5.2	4.4 ± 1.0	14.6 ± 0.8	14.6 ± 1.0	56.2 ± 1.1	70.7 ± 1,0
DEFL1-20_65-82_	>100	>100	−	−	−	−	−	−	−
DEFL1-23_65-82_	46 ± 2.7	56.7 ± 7.1	−	−	39.8 ± 3.2	−	57.1 ± 1.2	−	−
DEFL1-32_55-68_	97.8 ± 11.2	89.3 ± 7.2	−	−	42.9 ± 3.5	>100	38.5 ± 1.1	−	−
DEFL1-36_65-82_	38.3 ± 3.5	52.4 ± 4.3	>100	>100	16.8 ± 3.0	>100	48.6 ± 2.1	−	−
DEFL1-40_65-82_	59.3 ±5.1	49.9 ± 9.4	−	−	39.0 ± 1.2	−	51.6 ± 1.2	−	−
DEFL4-8_82-94_	−	−	−	>100	−	−	−	−	>100
DEFL4-20_86-110_	>100	>100	−	−	>100	−	>100	−	−
DEFL4-37_90-102_	−	−	−	>100	>100	>100	−	>100	−
HvDEFL4-1_67-77_	>100	−	−	>100	>100	>100	−	−	−
Tk-AMP-K2_10-23_	>100	−	>100	>100	>100	>100	>100	>100	>100
LTP2.25_50-62_	>100	−	−	−	45.0 ± 1.6	−	94.6 ± 3.7	−	−
TkSN1_39-57_	27.5 ± 1.2	93.6 ± 3.1	−	−	6.0 ± 1.5	164.7 ± 5.4	12.0 ± 2.3	−	−

«−» not determined.

## Data Availability

Not applicable.

## Supplementary Materials

The following supporting information can be downloaded at: https://www.mdpi.com/article/10.3390/ijms23158383/s1.
