# Supplementary figures and images for: The γ-Core Motif Peptides of AMPs from Grasses Display Inhibitory Activity against Human and Plant Pathogens
Source: Int J Mol Sci. 2022 Jul 29;23(15):8383. doi: 10.3390/ijms23158383 (PMC9368981; doi:10.3390/ijms23158383)

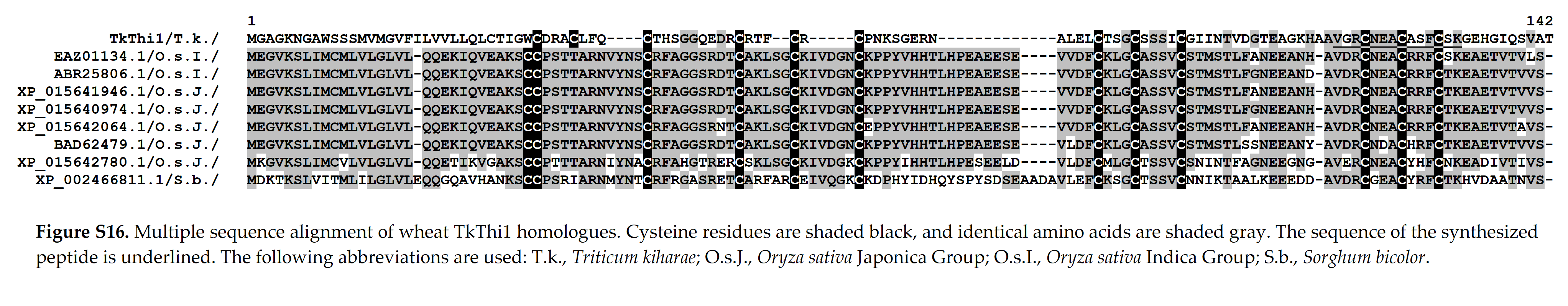

Supplement: Supplementary file 1 [file ijms-23-08383-s001.zip › Figure S16.tif]

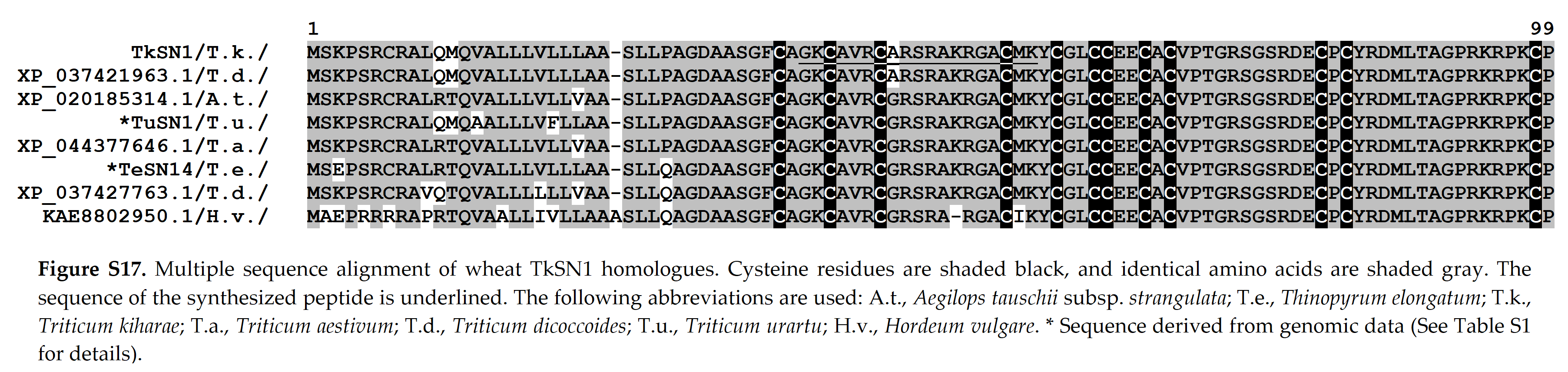

Supplement: Supplementary file 1 [file ijms-23-08383-s001.zip › Figure S17.tif]

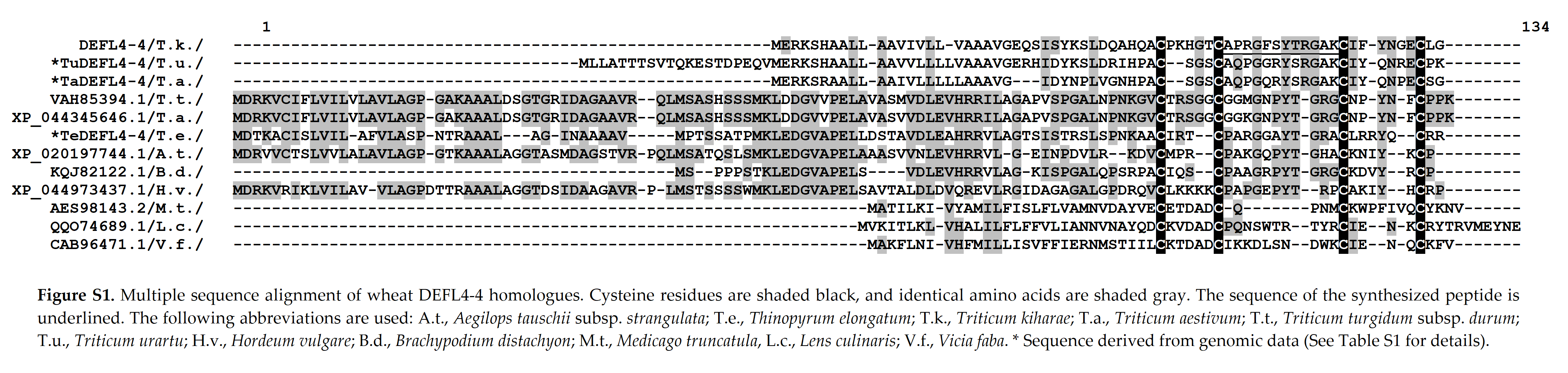

Supplement: Supplementary file 1 [file ijms-23-08383-s001.zip › Figure S1.tif]

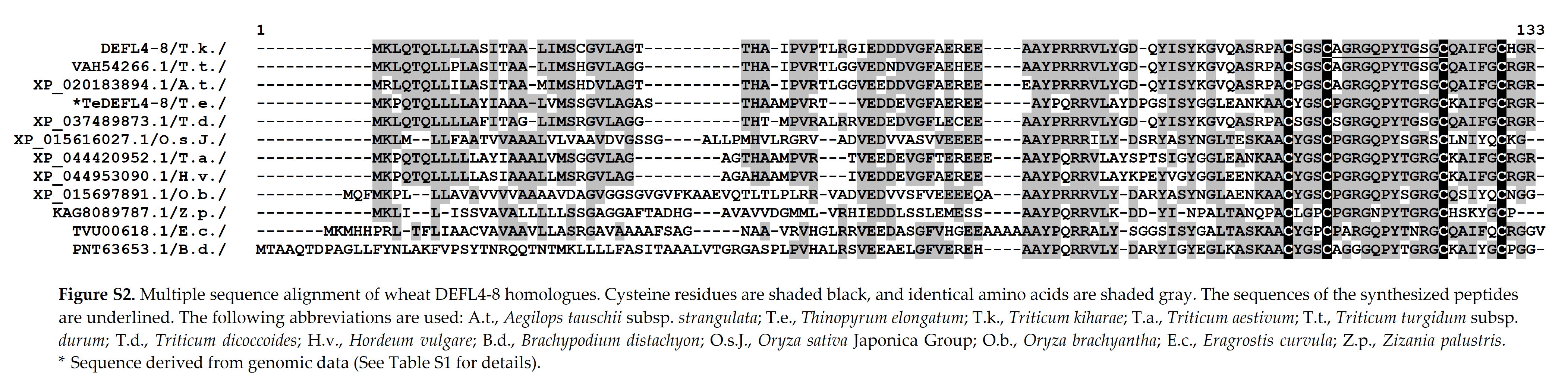

Supplement: Supplementary file 1 [file ijms-23-08383-s001.zip › Figure S2.tif]

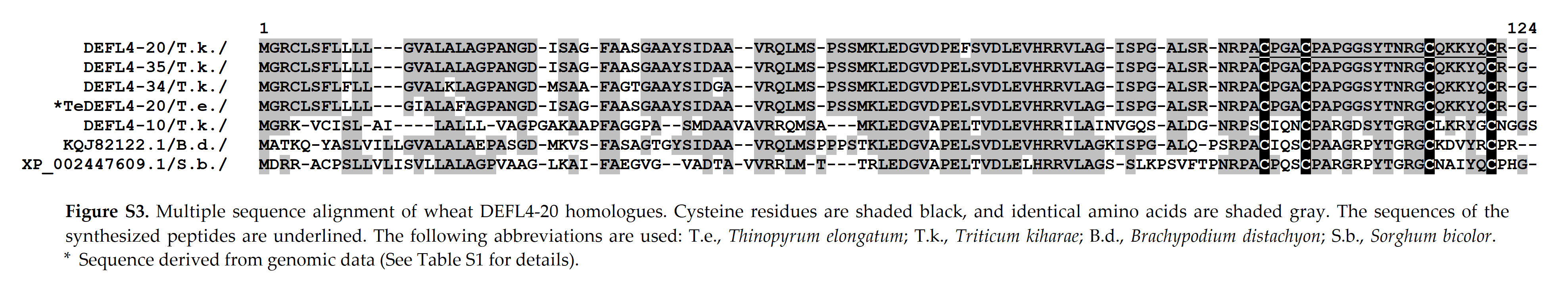

Supplement: Supplementary file 1 [file ijms-23-08383-s001.zip › Figure S3.tif]

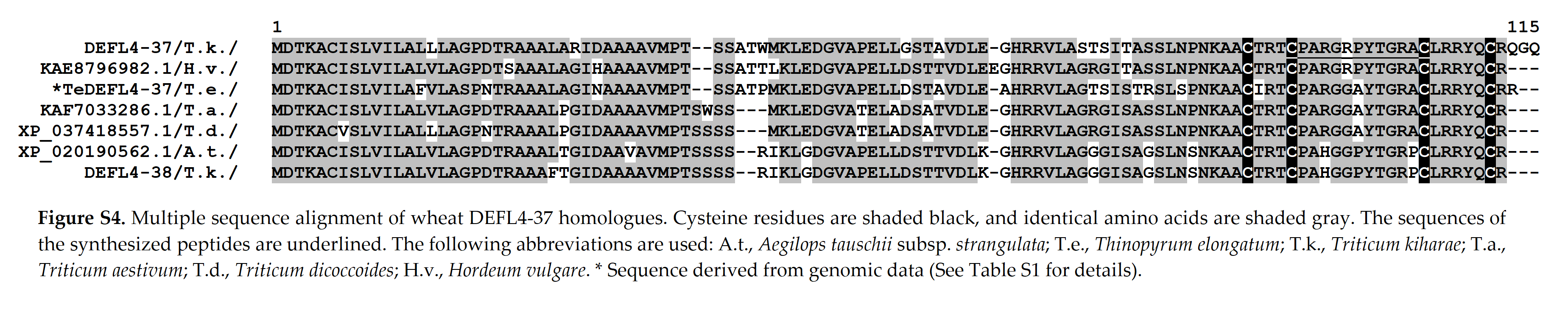

Supplement: Supplementary file 1 [file ijms-23-08383-s001.zip › Figure S4.tif]

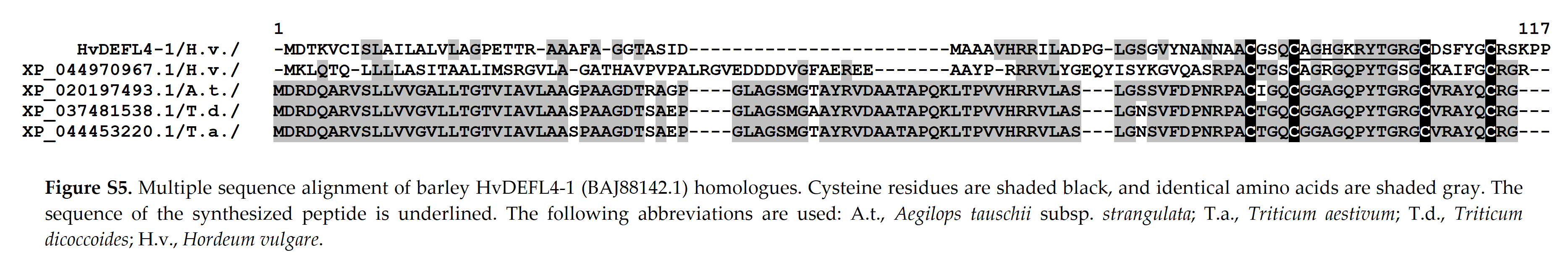

Supplement: Supplementary file 1 [file ijms-23-08383-s001.zip › Figure S5.tif]

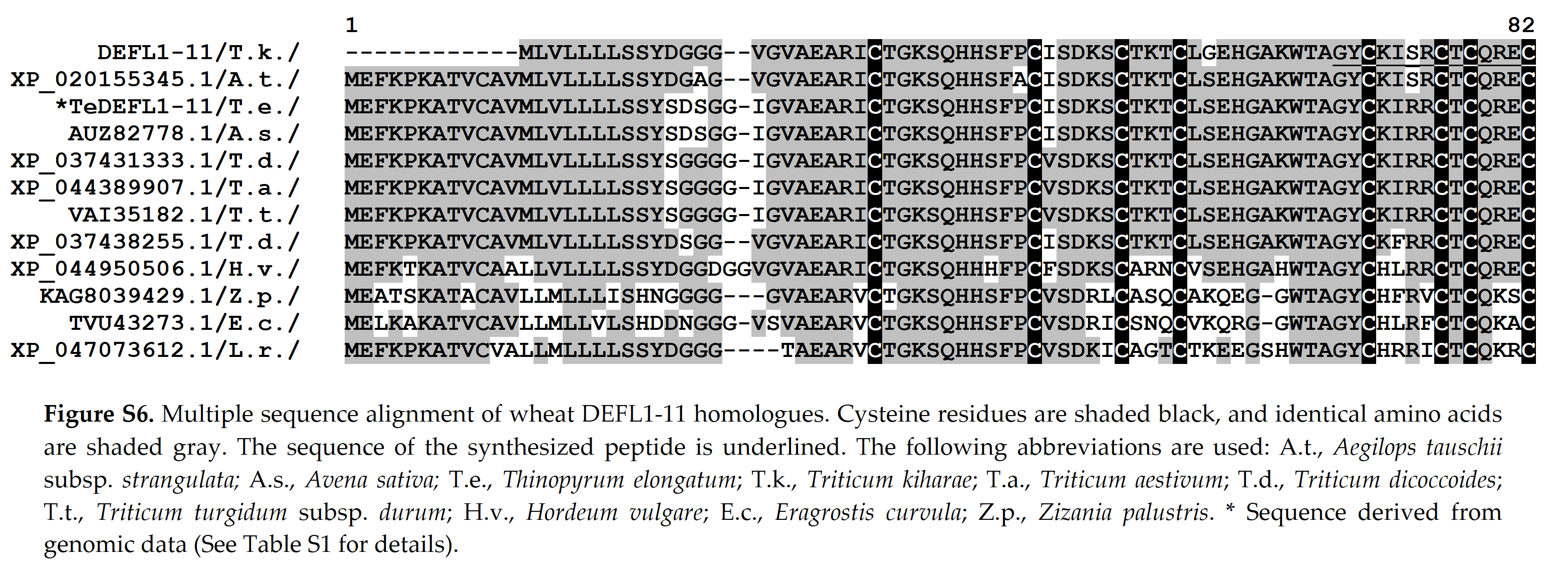

Supplement: Supplementary file 1 [file ijms-23-08383-s001.zip › Figure S6.tif]

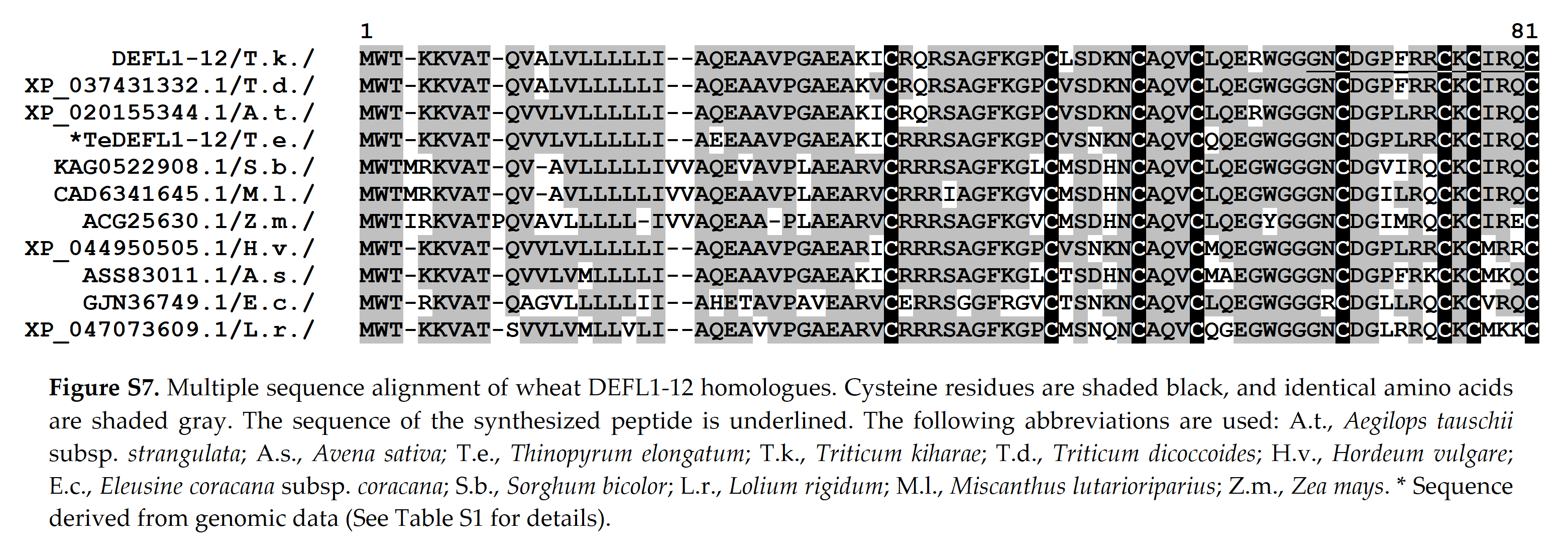

Supplement: Supplementary file 1 [file ijms-23-08383-s001.zip › Figure S7.tif]

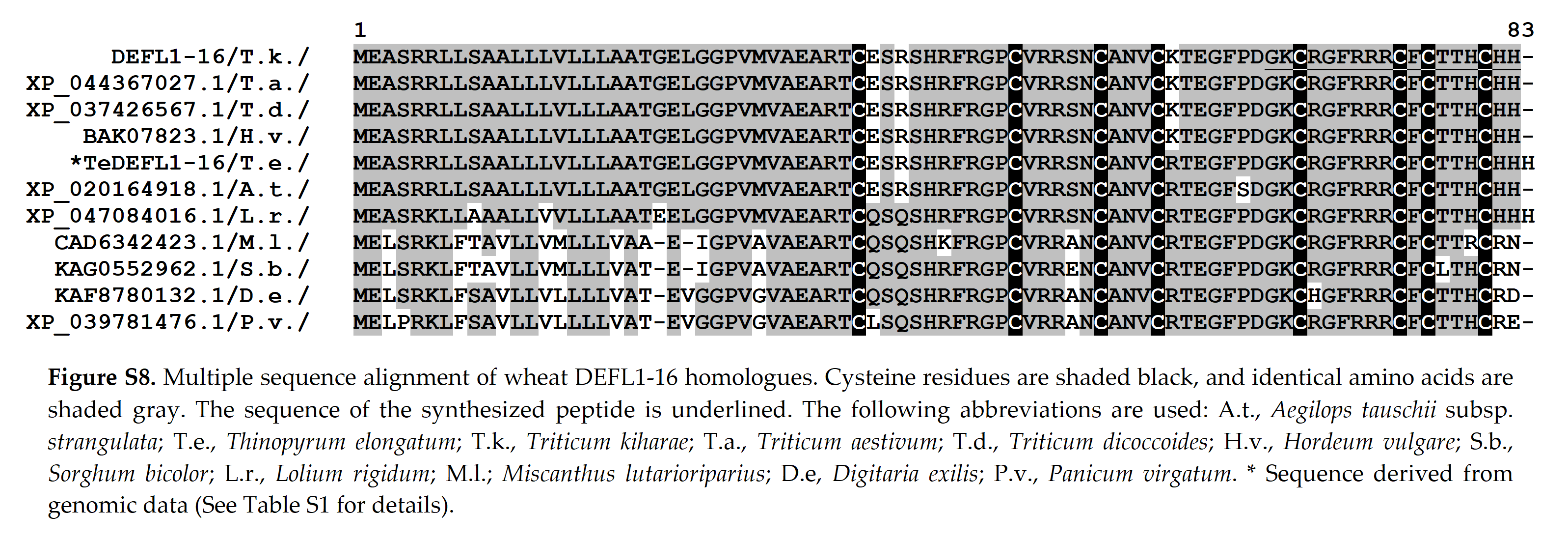

Supplement: Supplementary file 1 [file ijms-23-08383-s001.zip › Figure S8.tif]

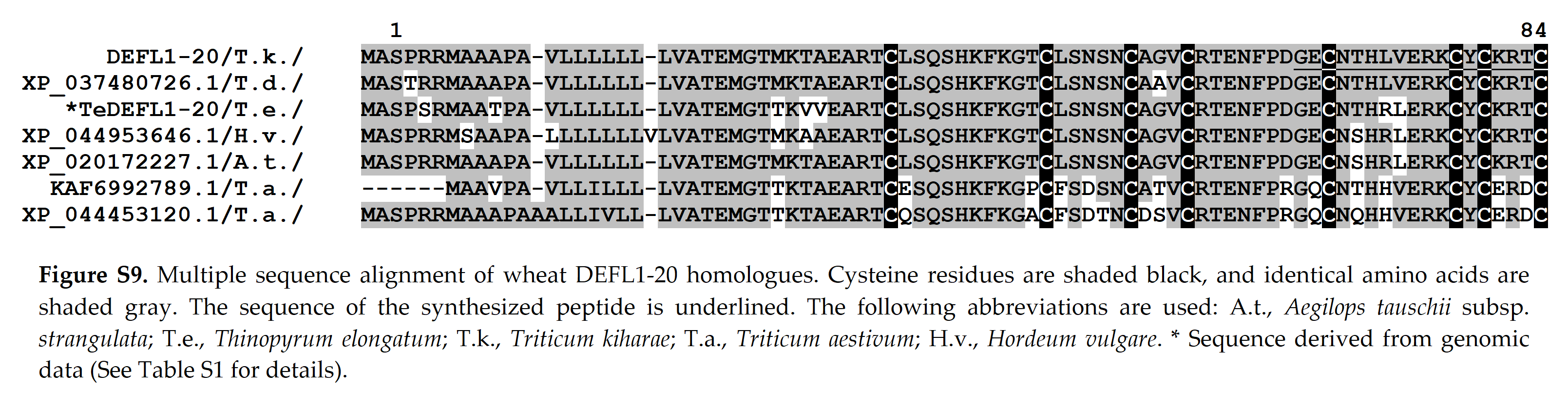

Supplement: Supplementary file 1 [file ijms-23-08383-s001.zip › Figure S9.tif]

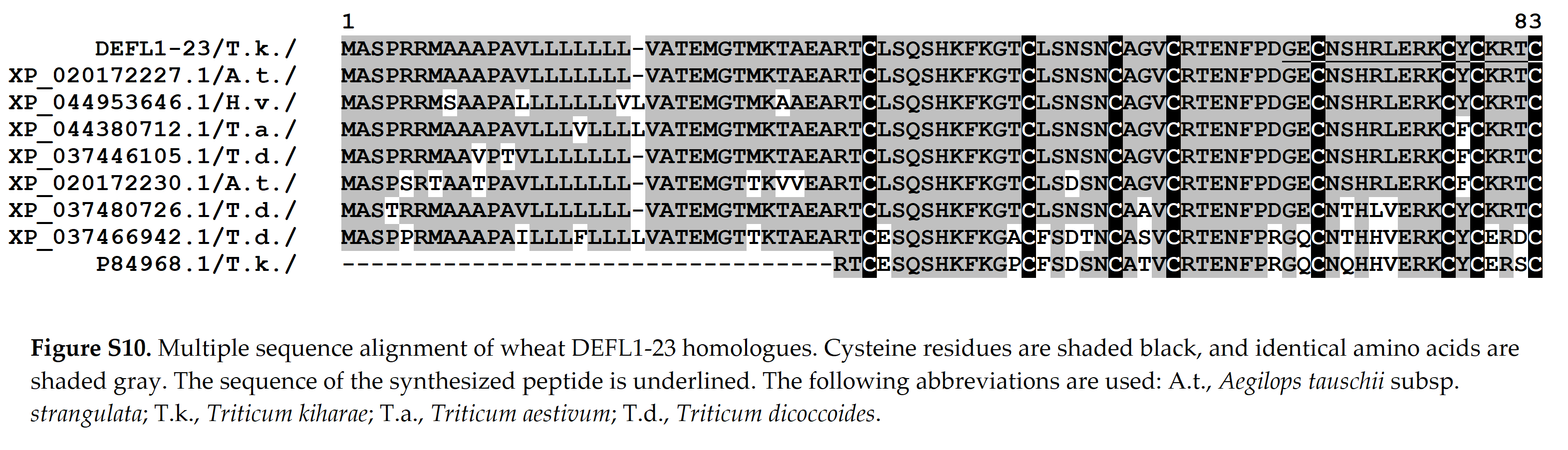

Supplement: Supplementary file 1 [file ijms-23-08383-s001.zip › Figure S10.tif]

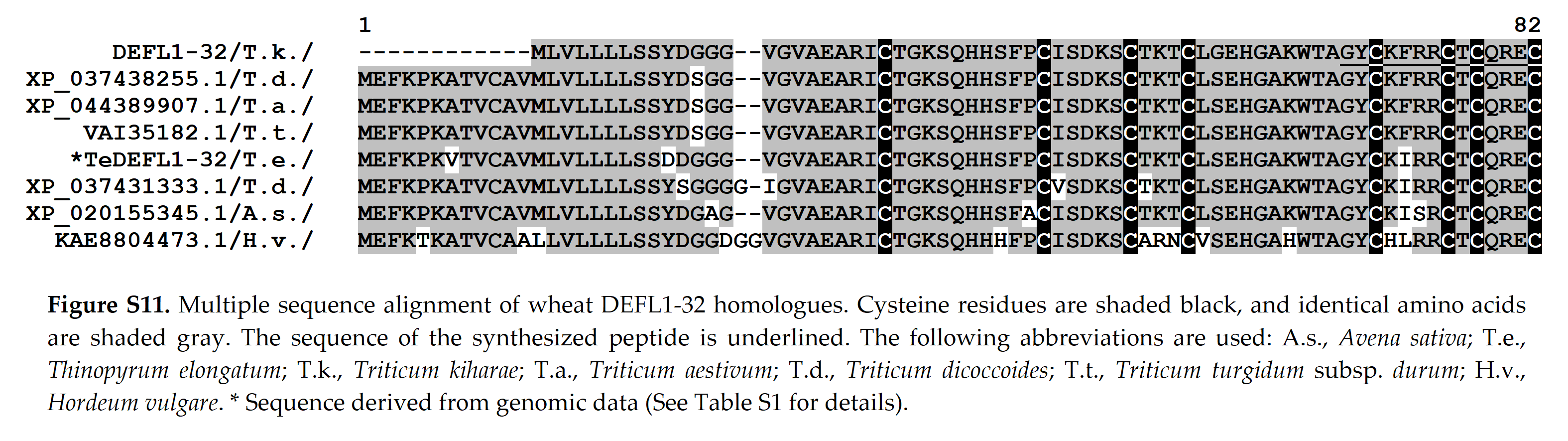

Supplement: Supplementary file 1 [file ijms-23-08383-s001.zip › Figure S11.tif]

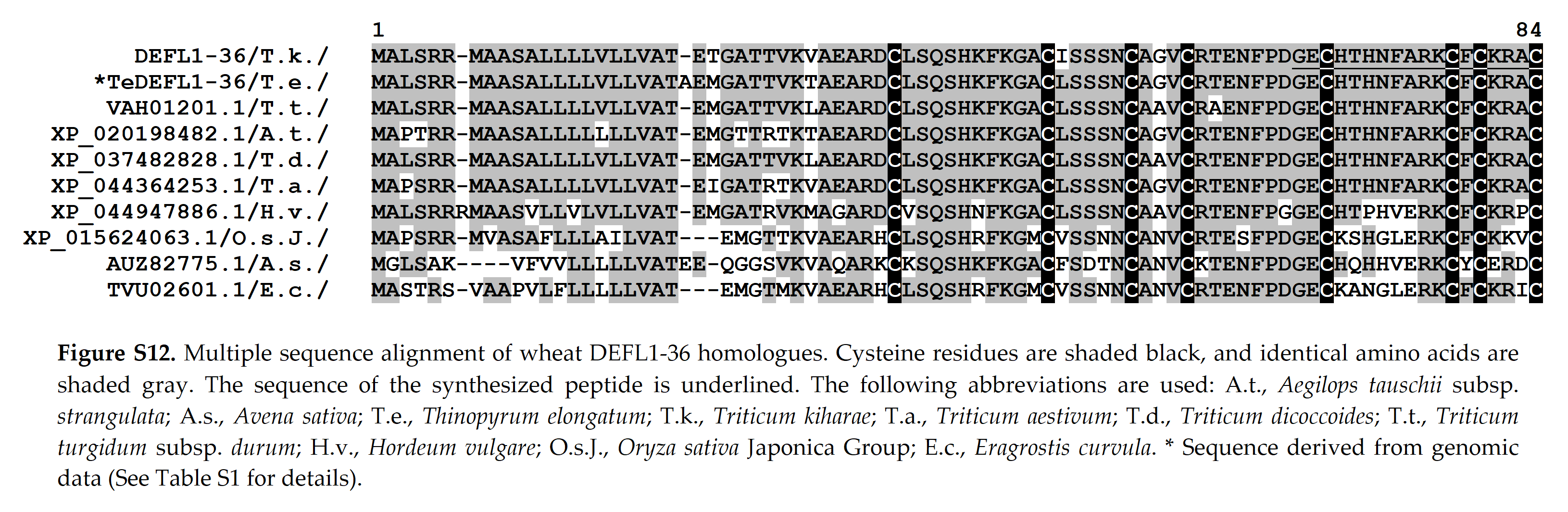

Supplement: Supplementary file 1 [file ijms-23-08383-s001.zip › Figure S12.tif]

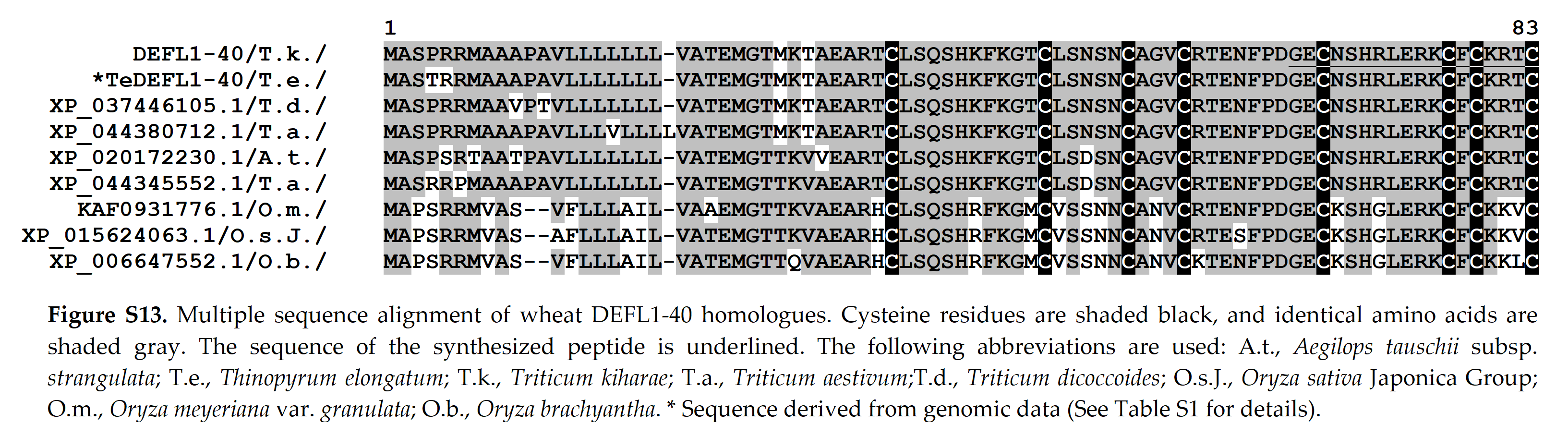

Supplement: Supplementary file 1 [file ijms-23-08383-s001.zip › Figure S13.tif]

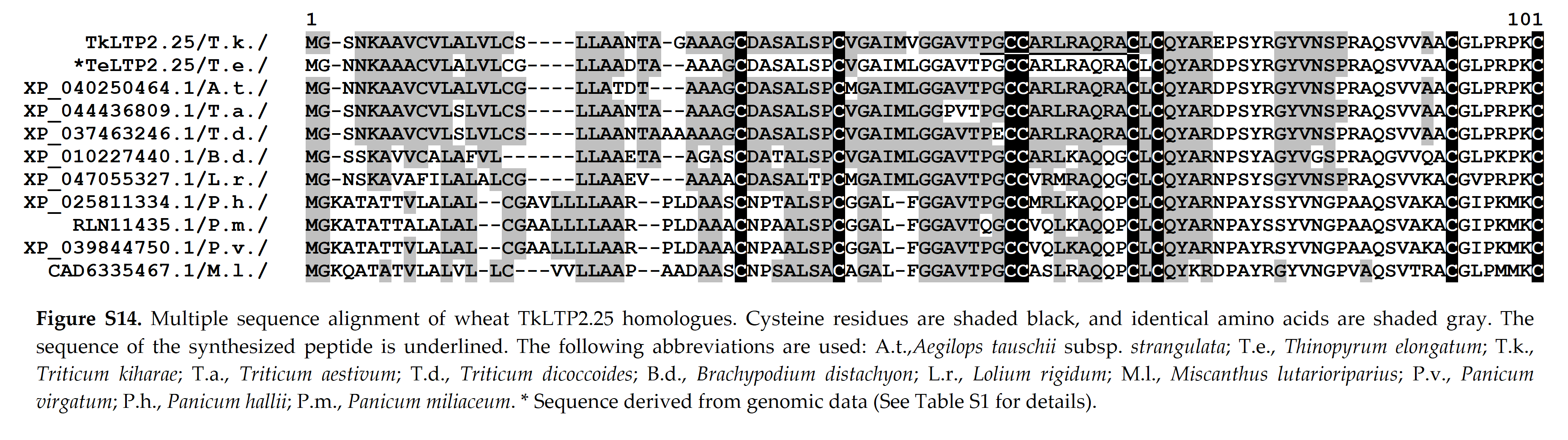

Supplement: Supplementary file 1 [file ijms-23-08383-s001.zip › Figure S14.tif]

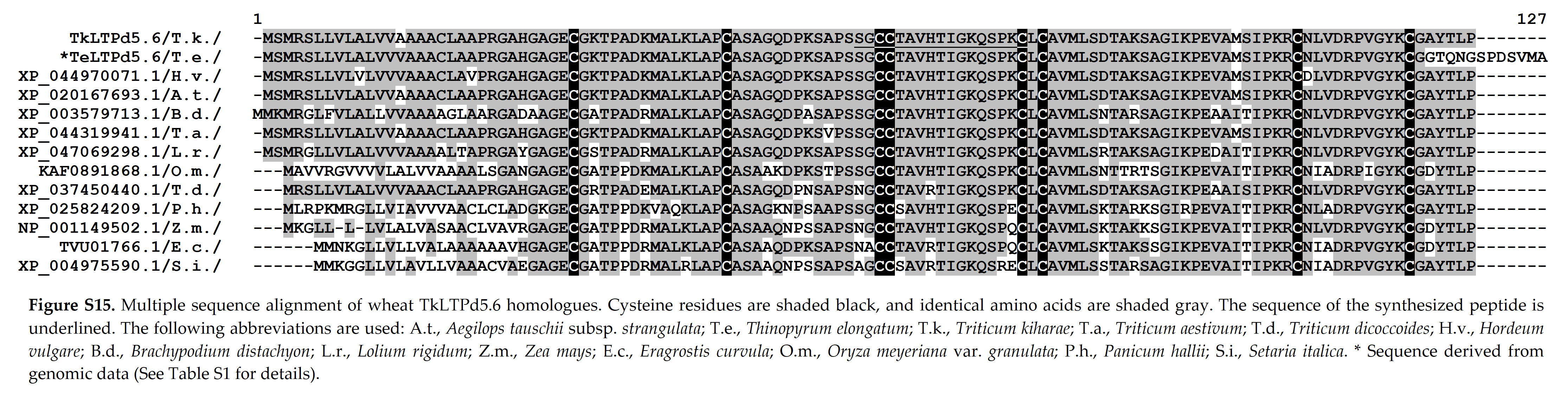

Supplement: Supplementary file 1 [file ijms-23-08383-s001.zip › Figure S15.tif]
